# Generative design optimization of tree distribution for enhanced thermal comfort in communal spaces with special reference to hot arid climates

**DOI:** 10.1038/s41598-025-96763-4

**Published:** 2025-05-13

**Authors:** Ahmed Maged, Aly Abdelalim, Abdelaziz Farouk A. Mohamed

**Affiliations:** 1 Landscape Architect, AE7, Cairo, EG Egypt; 2 Department of Architectural Engineering & Environmental Design, Arab Academy for Science, Technology and Maritime Transport, Cairo, EG Egypt

**Keywords:** Landscape parameters, Thermal comfort performance, Visual comfort, Communal spaces, Design optimization, Generative design tools, Civil engineering, Computational science, Urban ecology, Environmental impact

## Abstract

The quality of the communal outdoor environment is crucial for enhancing the urban quality and the well-being of its residents. These spaces are essential for providing more opportunities for social interaction and leisure. However, in hot arid climates like Egypt, achieving optimal outdoor thermal comfort remains a challenge. Accordingly, more comprehensive methodologies are highly needed to improve the research-based design of landscape parameters and components for developing outdoor thermal comfort performance using an iterative design exploration process that employs AI-driven software. These applications, help designers in solving multi-objective design quandaries through the generation and evaluation of numerous design options. Therefore, this study explores the efficiency of generative design tools in optimizing tree distribution based on mutation evolution to enhance outdoor thermal comfort, providing a dynamic, iterative approach that adapts to diverse urban morphologies. The methodology adopts a simulation-based analysis for framing this study, which is classified into three main phases. Firstly, analyze the current environment for specific outdoor spaces with different settings in Madinaty, New Cairo (fully clustered with buildings neighborhood, semi-clustered neighborhood, fully open neighborhood). Secondly, a generative design tool with a Dynamo evolutionary algorithm is utilized to optimize the tree distribution across the communal areas of these three spaces considering the current built environment. Lastly, testing thermal comfort using Grasshopper and Ladybug simulation to assess the Universal Thermal Climate Index (UTCI) between the base case scenarios and the optimized scenarios to validate the generative design tool. Results indicate tangible improvements across the three different neighborhoods. In the Clustered Neighborhood area, the optimized design with 33 trees resulted in a lower UTCI (with an arithmetic mean of 37.55 °C) compared to the base case with 43 trees (38 °C). In the Semi-Clustered Neighborhood area, the optimized design with 45 trees highly improves the UTCI (38.01 °C), compared with the base case with 27 trees (39.40 °C). Lastly, for the Fully Open Neighborhood area, the optimized design with 25 trees achieved a slightly improved UTCI (39.55 °C) over the base case of 31 trees (39.60 °C).

## Introduction and research background

The primary aim and focus of this research is to establish a methodology and framework that adapts generative design tools based on constraint-driven optimization logic, focusing on tree distribution strategies that enhance thermal comfort in communal spaces. Unlike traditional parametric models, generative design allows for continuous spatial refinement, optimizing vegetation placement based on environmental performance. Building on findings by^1^(Karimi et al., 2020) regarding vegetation’s role in thermal regulation, this approach explores multiple design iterations to identify the most effective configurations. Additionally, recent studies also show that integrating AI and genetic algorithms can improve environmental design optimization, allowing for more precise adaptation to site-specific climatic conditions^2^.

### Outdoor design measurements

Cities can benefit from comfortable urban outdoor spaces in several ways. One of the confirmed effects is the rise in usage of communal public outdoor areas which encourage socializing and community engagement^3^. As a result, these active forms of engagement improve physical health by increasing individual energy expenditures^4^. Numerous researches have demonstrated a positive correlation between effective urban & landscape design and public health^5^. Comfortable outdoor gathering spaces not only offer opportunities for interaction and socializing, but also have spiritual advantages. Various studies have elucidated this process, demonstrating that walking and other physical activities improve health and wellness and reduce the risk of disease and premature death^6^.

Meanwhile, the quality and conditions of urban environments also influence life in large and dense cities^7,8^. Additionally, comfortable outdoor spaces generally improving cities in terms of physical, environmental, economic, and social aspects^9^. Unfortunately, as a result of intensifying heat stress episodes caused by global warming, using outdoor spaces is becoming less common and limited^10^. Climate change projections indicate dangerously long durations of discomfort, which can have detrimental effects on people’s health and well-being^11^. In addition to climate change, future urbanization is expected to result in more discomfort hours in cities due to vicissitudes in land use and anthropogenic heat emissions^12^.

Thus, in order to create comfortable urban outdoor spaces, it is crucial to adopt optimal urban design. One of the design strategies that can be used to improve comfort levels in outdoor spaces is tree planting and proper distribution across the entire space^13^. Based on previous research, green spaces can enhance the urban microclimate by increasing shading and evapotranspiration, which lowers longwave exchange and heat gains while increasing latent cooling, consequently positively improving thermal comfort^14^. Additionally, vegetation can influence acoustic comfort, further enhancing the entire environmental quality of urban spaces^15^.

Additionally, a lot of research on green infrastructure has been carried out to lessen the effects of urban heat islands (UHIs), which are worsened by increasing urban density^16^. Recently, the concept of green spaces as nature-based solutions (NbSs) has gained prominence once more in response to the challenges posed by climate change^17^. The potential of trees to mitigate the effects of climate change is being increasingly studied in light of this^18^. It has been concluded that one of the most important ways to counteract the impact of UHI and adapt to climate change is to implement green infrastructure around residential buildings.

In these terms and since the importance of outdoor spaces’ environmental performance, the study of the microclimate and thermal comfort in outdoors has become an important topic in the field of landscape architecture. The majority of existing research on urban microclimates is categorized by landscape elements, such as squares, grass, tree-shaded spaces, watersides, etc., However, this classification does not comprehensively consider the superposition and distribution effects caused by landscape elements or how they can optimize thermal comfort.

### Outdoor thermal comfort (OTC)

**Fig. 1:**
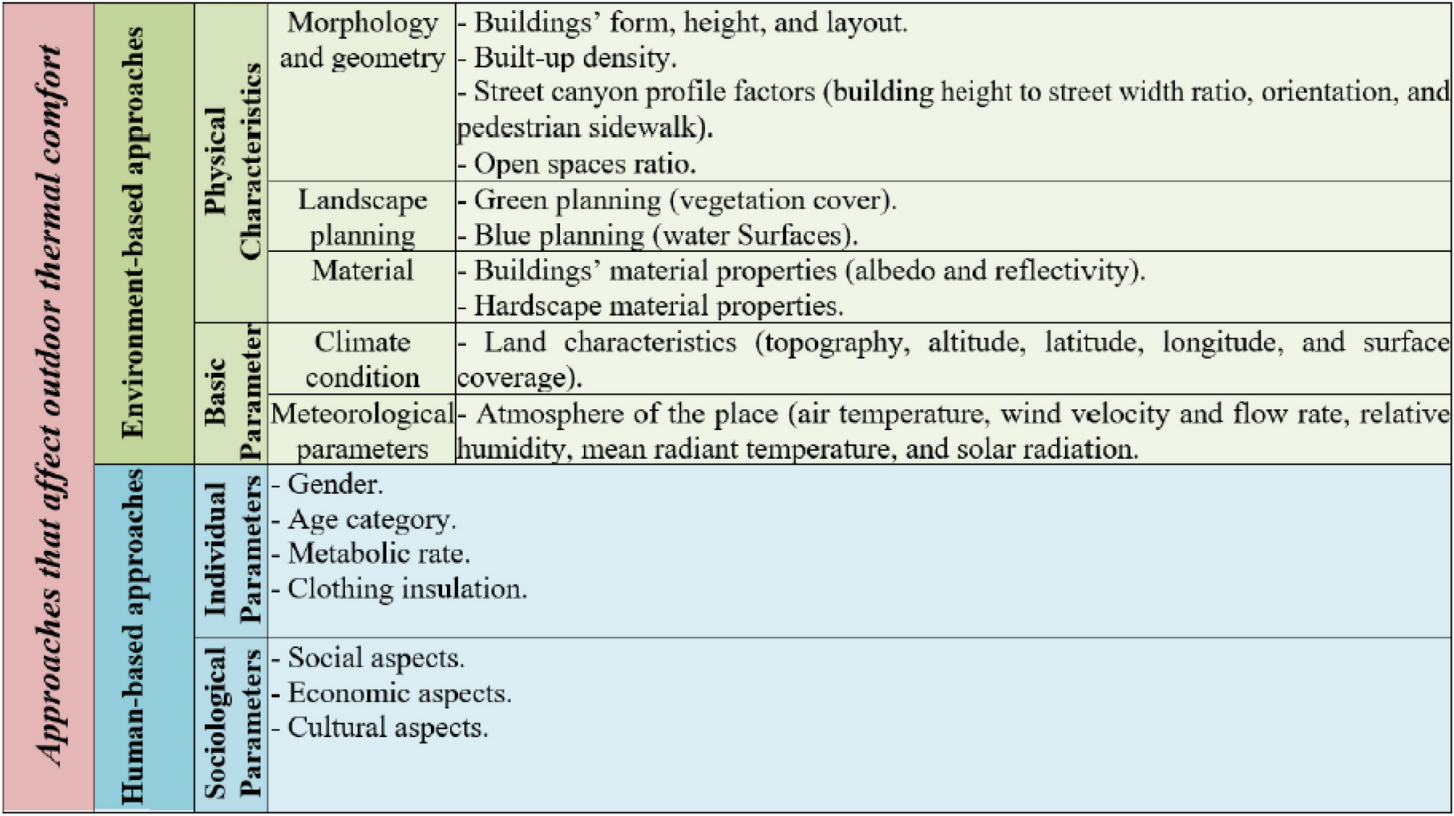
Approaches that affect the OTC, by (El-Bahrawy, A., 2024)^21^.

In addition, thermal comfort can be characterized as a mental state of mind expressing contentment with the ambient thermal conditions. It is a primary measure of the quality of an urban area in regard to outdoor areas; ensuring suitable thermal comfort prolonging the time that people can spend outside and enhancing the city’s life^19,20^. Nonetheless, outdoor thermal comfort (OTC) is affected by various disciplines, and researchers have studied and categorized these components in several studies; (Fig. 1 summarizes most of them)

When assessing OTC, the designers had to deal with the complexity of managing all these components. In light of this, numerous researchers have worked to standardize thermal comfort over the past few decades by developing thermal comfort tools and indices; among these indices and tools were Predicted Mean Vote (PMV), Physiological Equivalent Temperature (PET), and Universal Thermal Climate Index (UTCI)^22^. Since the research has used the extremely hot week of the year as an experimental Duration, UTCI was used to assess the OTC of the design and outputs derived from the simulations of the generative design tool to properly validate the tool. Additionally, to assess how the optimization of the tree distributions and coverage percentage affects the UTCI values.

### Universal thermal climate index (UTCI)

**Fig. 2:**
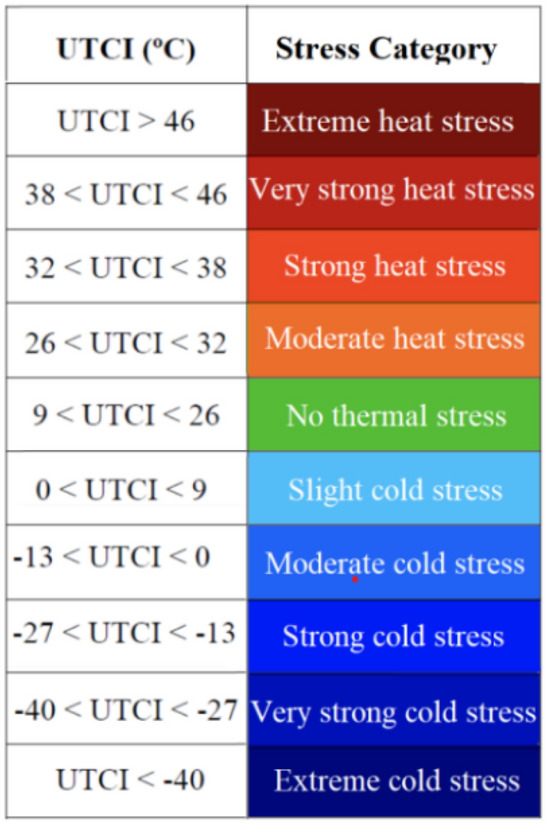
Equivalent temperature scale and stress category by P. Bröde et al., 2011)^24^.

Furthermore, the International Society on Biometeorology (ISB) and the Cooperation in Science and Technical Development (COST-Action 730), which are eminent authorities in the fields of human thermophysiology, physiological modeling, meteorology, and climatology, collaborated internationally to develop UTCI in 2009. Their goal was to develop an index that would serve as an international standard for describing the effects of meteorological climatic conditions (air temperature, radiation, air humidity, and wind speed) and thermophysiological factors that affect humans (clothing and activity). Accordingly, the UTCI is defined as the air temperature (Ta) of the reference condition causing the same model response as actual conditions^23^. It simply explains the thermal stress caused by the interaction of air temperature, mean radiant temperature, relative humidity, wind speed, and thermophysiological and behavioral clothing models on an equivalent temperature scale^25^. Based on criteria derived from the simulated physiological responses, the UTCI values were classified into ten categories (Fig. 2) of thermal stress, ranging from"extreme cold stress“to”extreme heat stress".

Furthermore, the advanced multi-node model of thermo-regulation by (Fiala et al., 2012)^26^ serves as a basis for the UTCI. The ability of an organism to maintain its body temperature within predetermined ranges despite drastically different environmental temperatures is known as thermoregulation (see equation below, where **Ta (°C)** is the Temperature, **Tmrt (°C)** is the mean Radiant, ${\varvec{\nu}}$ **(m/s)** is the wind speed and *e* **(Pascale Pa)** is the water vapor pressure).

$$UTCI\sim \mathcal{F}(Ta, Tmrt, \nu ,e)$$

**Fig. 3:**
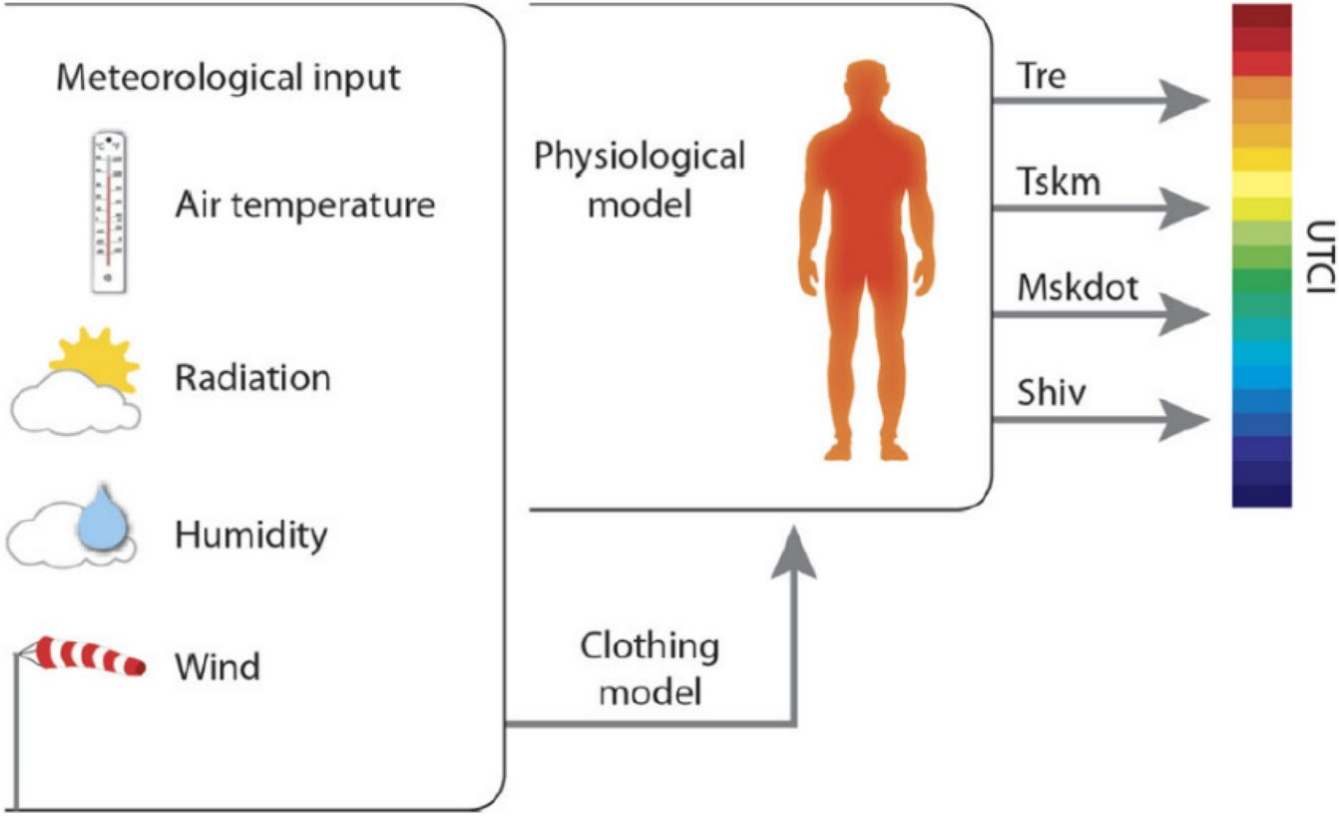
Concept of the UTCI derived from the dynamic multivariate response of the thermo physical UTCI-Fiala model which was coupled with a clothing model. **Tre** rectal temperature, **Tskm** mean skin temperature, **Mskdot** sweat production, **Shiv** heat generated by shivering.

Fiala’s model is combined with (Havenith et al., 2012)^27^ advanced clothing model, which accounts for the **Ta**-driven behavioral adaptation of clothing insulation in the general public (Fig. 3, by Bröde et al., 2013)^25^. The UTCI is conceptually derived as an equivalent temperature. For any combination of **Ta**, **Tmrt**, **v**, and **e**, UTCI is defined as the air temperature that would provoke the same dynamic physiological response (strain) under reference conditions.

### Generative design tools and system deployment

While prior studies by (Karimi et al., 2023; Halder et al., 2022)^28,29^have analyzed the impact of urban vegetation on thermal performance, this study advances the field by employing a generative design framework using constraint based design logic to optimize tree distribution dynamically, ensuring adaptability across different urban settings. Furthermore, integrating artificial intelligence into the landscape design process can provide a chance to resolve multi-objective design dilemmas. Generative design is considered a form of AI integrated into the landscape, involving the cooperation of technology and human creativity. It is based on, Humans inputting the objectives and constraints of the design issues, and the computer, undertaking various automated generations, assessments, and mutation based evolutions, produces hundreds of high-performing solutions and selects the optimum solution^30^.

Noting that the approach itself is not restricted to any specific area of application, even though there are currently areas where generative design is applied and developed more actively. Currently, generative design is frequently utilized in the industrial design, graphic design, and web design fields. Recently, generative design tools have been introduced into the construction field sector and have become one of the most promising areas for improving modern building information modeling (BIM) systems. In the field of landscape design, visual programming editors and tools such as Rhinoceros 3D Grasshopper and Autodesk Revit, which are compatible with BIM systems Graphisoft Archicad and Autodesk Revit, respectively, are the most widely used tools for developing generative algorithms.

From this point of view, this study focuses on how tree positioning and distribution can be designed by algorithmic thinking tools while simultaneously considering the existing built environment and UHI. Furthermore, this will be further tested by using UTCI, which is considered one of the most widely used indices that has proven its efficiency in evaluating the OTC. Moreover, this methodology explores extensive design options to create numerous technically feasible solutions that may be beyond experts’ experience and cognitive capacity. Aiming to enhance outdoor thermal comfort and provide various solutions in the pre-concept design stages.

## Methodology

**Fig. 4:**
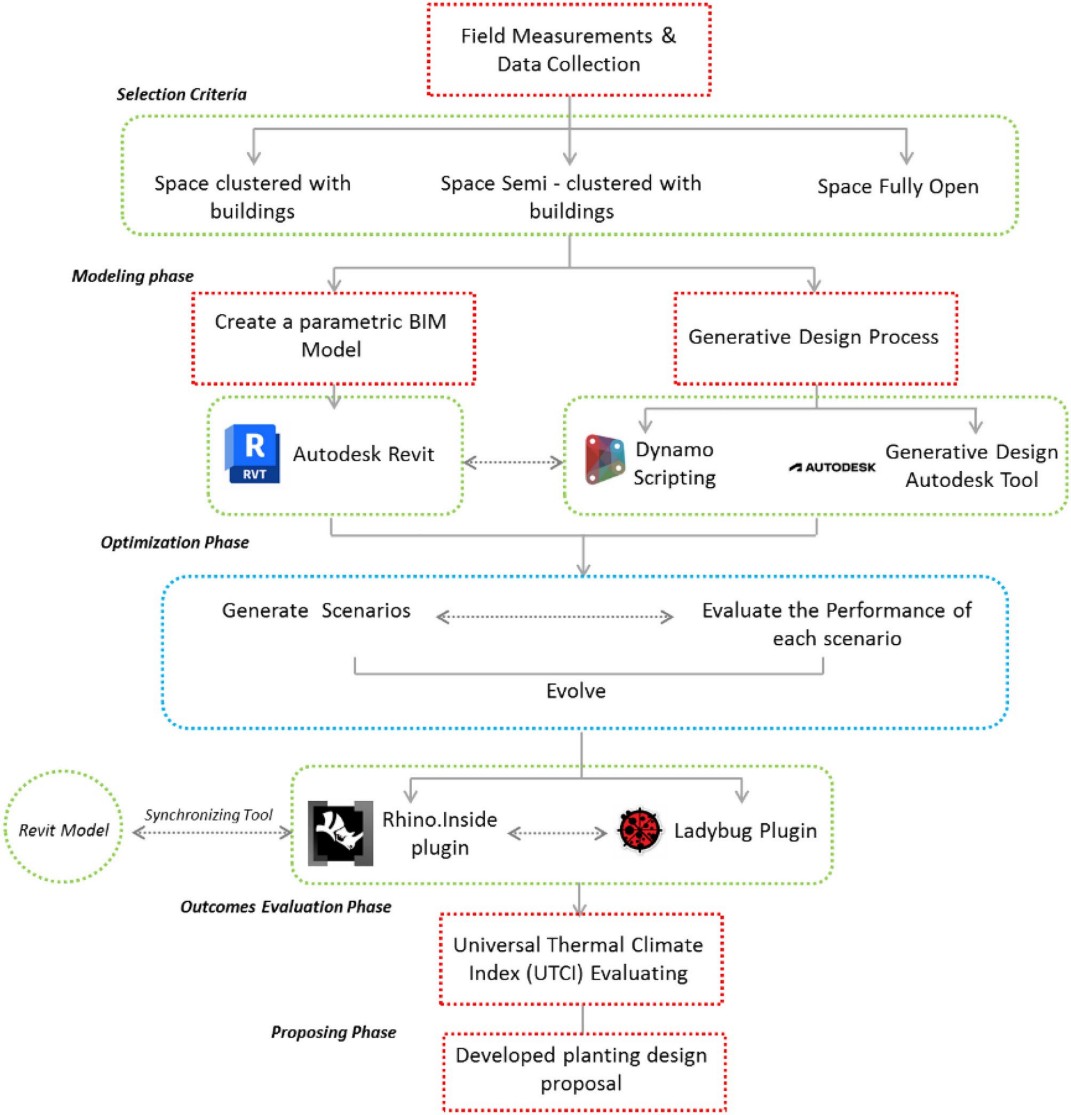
Methodology framework and research workflow by the Author.

This study employs an iterative AI-driven generative design process, utilizing mutation-based evolution and seed control techniques to refine tree placement dynamically based on environmental constraints and performance feedback, distinguishing it from conventional parametric methods. The evaluation unit in this study is the Universal Thermal Climate Index (UTCI), as it is mainly used to evaluate and analyze the overall OTC and provides a comprehensive indicator of outdoor thermal stress. (Fig. 4 shows the research framework).

### The methodology and workflow are composed of four main parts

- Firstly, three communal spaces with varying built environments and morphologies were selected for field measurements and data collection (neighborhood clustered with buildings, semi-clustered neighborhood, and fully open neighborhood). However, the intent of choosing three different cases is to have more accurate insights into the ability of generative design tools to work in various urban settings.
- Secondly, a parametric model is created for the three chosen urban settings using Revit 2024®. In addition, a dynamo script is developed by specifying the main inputs, constraints, and outputs, while also distinguishing the evaluators and the visualization procedures of the optimization tool. A multi-objective simulation study is then conducted using a generative design tool over a specific date and duration, examining hundreds of design options to determine an optimal scenario for tree distribution.
- Thirdly, thermal heat maps of the proposed models with different tree arrangement proposals were generated through simulations, revealing how the thermal environment changes under different scenarios while considering the surrounding built environment.
- Fourthly, generating the optimized scenario and utilizing Rhino.Inside.Revit v1.0® plugin to merge Autodesk Revit and Rhino into the modeling and simulation process. It primarily offers translation API to allow the creation of custom conversion workflows between the designed Revit data and Rhino geometry and metadata^31^.
- Lastly, a comparative analysis is conducted with the base case and the new optimized case in terms of UTCI through using Ladybug© plugin imported in Grasshopper in order to determine the effectiveness and synergy of the generative design tool.

### Examination and screening process

The examination process for the tree design is conducted by evaluating the UTCI of the best option across the entire population size of the scenarios. However, the selection is mainly based on selecting the option with the proper tree design that gives the highest coverage percentage and with minimal tree counts. In order to help decision-makers achieve delicate coverage with the lowest possible cost.

### Area of study

**Fig. 5:**
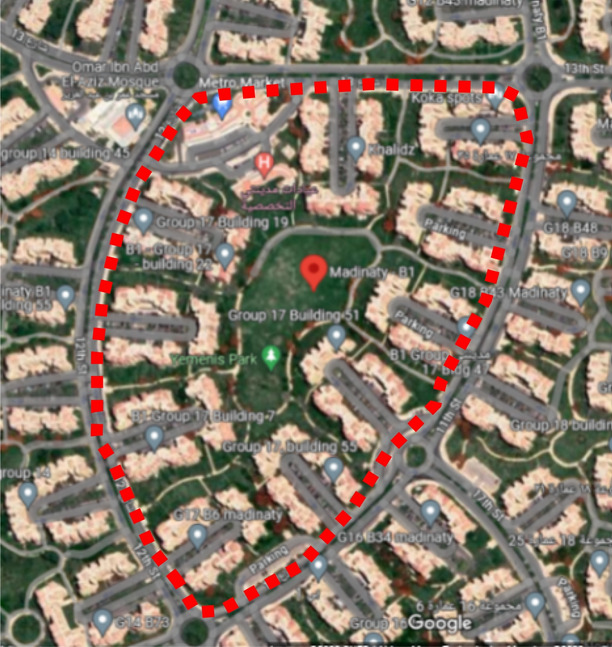
Fully Clustered Area, Image extracted from google maps^34^.

**Fig. 6:**
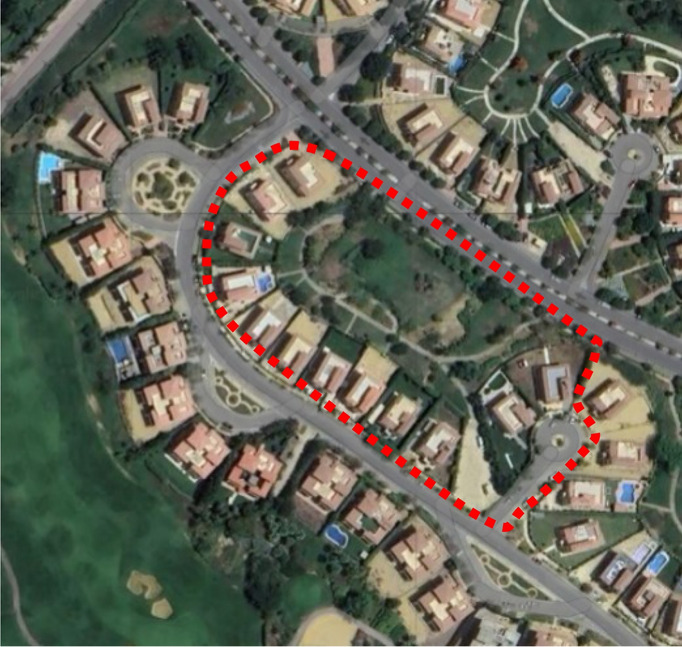
Semi-Clustered Area, Image extracted from google maps^34^.

**Fig. 7:**
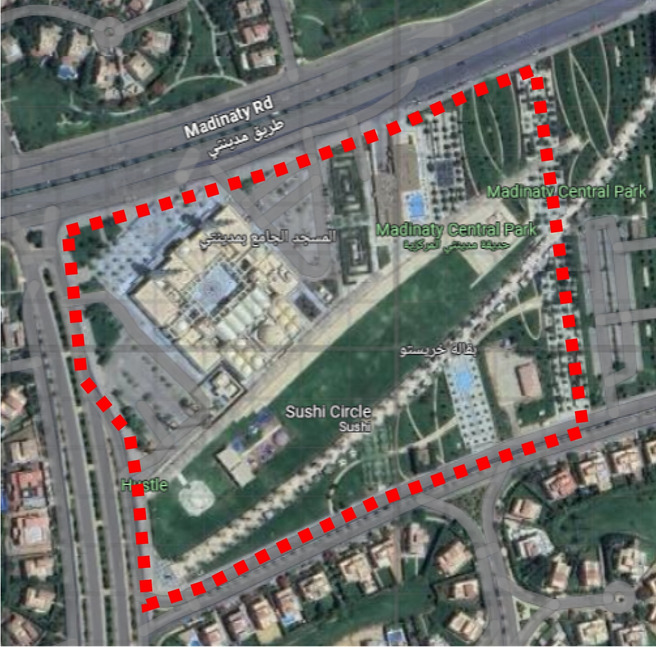
Fully open Area, Image extracted from google maps^34^.

The study areas chosen for the modeling and optimization process are in Madinaty which is considered an 8000-acre real estate development project, in the New Cairo satellite city, in the Eastern area of Cairo, Egypt. Madinaty, is one of the largest residential neighborhoods in Cairo. It is located, between 30.096° North Latitude and 31.662° East Longitude^32^. Madinaty takes attention from its dense urbanized structure with its vibrant and vivid neighborhood culture. In this respect, “Madinaty” offers the appropriate environmental and social significance for the model generation which offers a unique setting with a variety of urban configurations, including fully clustered (Fig. 5), semi-clustered (Fig. 6), and open space (Fig. 7). This diversity allows for a comprehensive examination and better validation of the generative design tool.

Moreover, concerning the usage of outdoor spaces, it can be observed that people use the in-between spaces and large green areas such as parks rather than viewing gardens. Although the presence of vegetative elements in the area encourages people to spend more time outside. Nonetheless, the spatial configuration and distribution of landscape components can be discussed. Considering that the average UV index value of Cairo is approximately 7 to 9 depending on the season, additionally, The annual average daily value for the clear sky index (Kt) is 0.566 and the annual average daily global irradiation value is 18.67 MJ/m^2^with the average temperature values reaches 28° degrees, accordingly, the results show that Cairo is characterized by relatively high average-daily radiation rates and temperature also, in general, Egypt’s climate according to the Koppen Climate Classification subtype is mostly hot arid Climate. Moreover, it was highly observed that the use of open space is related to the microclimatic structure of the area^33^.

#### Selection criteria for the three spaces

The selection of the three spaces is guided by diverse factors to ensure the adequacy of the study and variety in the outdoor urban environment for better tool validation (Table 1).

**Table 1:** Selection criteria for the three neighborhoods.

	Clustered with buildings	Semi-Clustered with buildings	Open space
Urban density	High density	Moderate density	Low density
Sun exposure and building shadows	Significant building shadows.	Partial sun exposure; mix of building shadows and sunlight	Full sun exposure; minimal shadows come from buildings
Thermal comfort challenges	High heat buildup	Mixed thermal conditions	Potential for overheating
Accessibility	Limited access points	Moderate accessibility	High accessibility

#### In-Depth analysis of key study areas

The assessment process mainly analyzing the existing conditions of the three main spaces to gain a better understanding of the context and ensure accuracy in the modeling phase. (Table 2)

**Table 2:** Data measurements and analysis.

	Clustered with buildings	Semi-Clustered with buildings	Open space
Total area of land (m^2^)	154,462 m^2^	33,853 m^2^	76,357 m^2^
Area of communal space (m^2^)	13,944 m^2^	5,634 m^2^	14,171 m^2^
No. of buildings (Nr)	55 Residential building	16 Residential building	1 Mosque + 1 commercial building
Height of buildings (m)	22 m	10 m	45 m
No. of existing vegetation (Nr)	37 trees + 6 palms	19 trees + 8 Large shrubs	31 palms

However, it was observed during the data collection phase, even in fully clustered neighborhood, where buildings offer substantial shading, users were still significantly uncomfortable due to a deficiency of trees and plantations. Insufficient vegetation placements in these areas frequently result in higher surface temperatures and decreased outdoor comfort, even with the large shadows cast by buildings. However, this will be further presented in the UTCI simulations.

Moreover, the inadequate tree cover did not properly moderate the impacts of direct sunshine and heat accumulation on surfaces, demonstrating that building shadows alone are insufficient to achieve ideal thermal comfort. In light of that, this can raise the importance of including additional green infrastructure, and a proper distribution of trees, to improve shading and cooling benefits in different urban settings.

#### Existing vegetation analysis and classifications

The existing vegetation is being analyzed with an emphasis on determining the types, densities, and distributions of the plant species that currently exist in each neighborhood. As part of this analysis, vegetation is categorized according to different factors such as height, species type, and canopy cover. This helps ensure accuracy during the modeling phase of the base case scenario and provides a general overview of the types of vegetation and their characteristics in each study area to be followed later in the optimization phase.

- **Clustered neighborhood area**

**Fig. 8:**
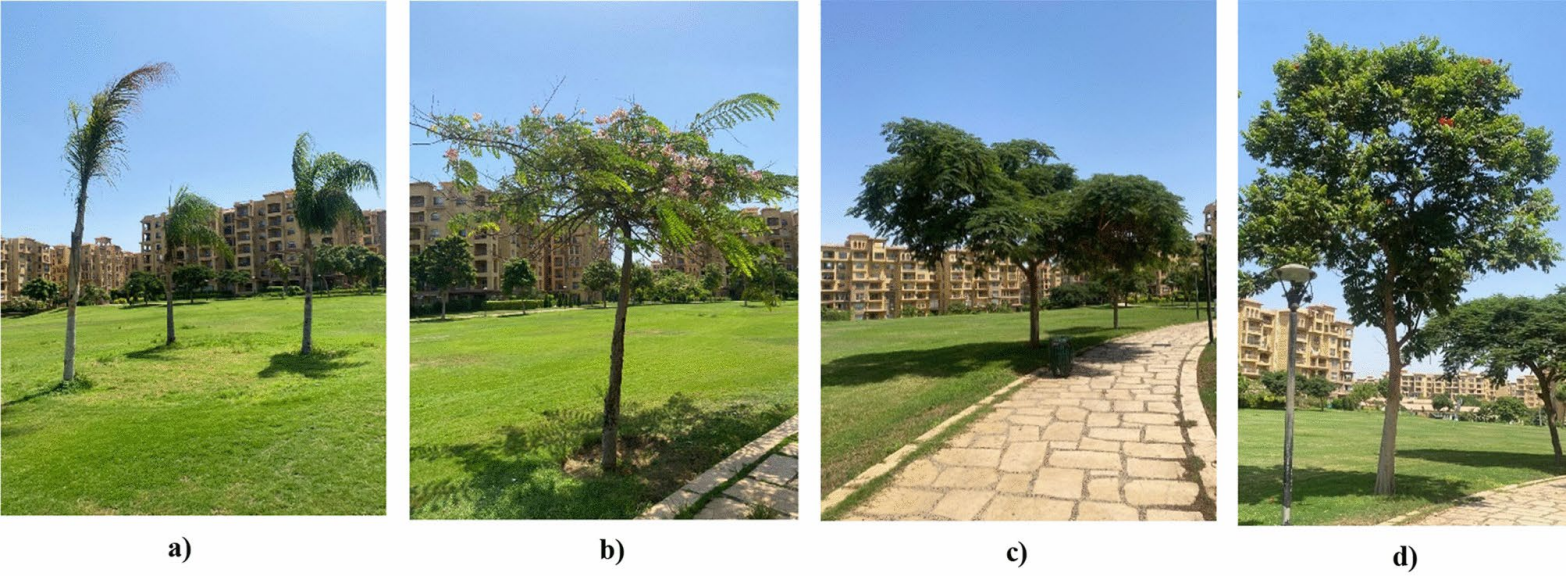
Clustered Neighborhood Vegetation design, Site images taken by the Author. **a** Syagrus Romanzoffiana Palm. **b** Cassia Nodosa Tree. **c** Poinciana Regia Tree. **d** Spathodea Campanulata Tree.

For the clustered enclosed area, approximately 37 trees outline the entire boundary of the communal space, and 6 palms are centralized in two groups of 3 palms at the center of the boundary. Additionally, a mix of adaptive mature trees that were analyzed in terms of height, type, and canopy width. (Fig. 8), (Table 3).

**Table 3:** Clustered neighborhood plant’s size and form^35^.

	**Form**	**Height**	**Canopy width**	**Total number**
Saygrus romanzoffiana palm	Small-trunked, tall palm	10–13 m	6–7 m	6 Palms
Cassia nodosa tree	Tree with an umbrella-shaped crown	10–15 m	6–8 m	3 Trees
Poinciana regira tree	Many horizontal large branches	10–12 m	6–8 m	13 Trees
Spathodea campanulata tree	Open leafy tree	10–15 m	5–10 m	21 Trees

b) **Semi-Clustered neighborhood area**

**Fig. 9:**
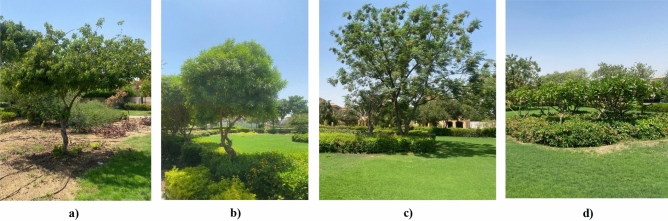
Semi-Clustered Neighborhood Vegetation design, Site images taken by the Author. **a** Cassia Glauca Tree. **b** Thevetia Neriifolia Tree. **c** Jacaranda Mimosifolia Tree. **d** Plumeria Alba Shrub.

There are 19 trees and 8 large shrubs scattered inside the boundary. (Fig. 9), (Table 4).

**Table 4:** Semi-clustered neighborhood plant’s size and form^35^.

	Form	Height	Canopy width	Total number
Cassia glauca tree	Rounded, small tree	3–6 m	2–3 m	6 Trees
Thevetia neriifolia tree	Erect, small tree with bright flowers	3–5 m	1.5–3 m	12 Trees
jacaranda mimosifolia tree	Large, spreading deciduous tree	10–15 m	7–10 m	1 Tree
Plumeria alba shrub	Deciduous, many-branched small	2–5 m	2–3 m	8 Shrubs

c) **Fully-open neighborhood area**

**Fig. 10:**
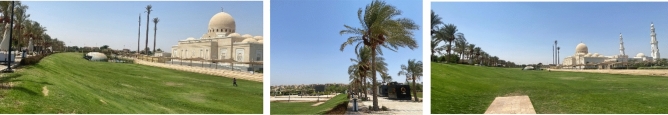
Phoenix Dactylifera Palm and the open green communal space, Site images taken by the Author.

There are only 31 Phoenix Dactylifera Palm alongside the communal area. (Fig. 10), (Table 5).

**Table 5:** Fully-open communal area plant’s size and form^35^.

	**Form**	**Height**	**Canopy width**	**Total number**
Phoenix dactylifera palm	Tall, columnar trunk	15–20 m	5–7 m	31 Palms

### Parametric modeling process and creation

The process starts with creating a digital model on Revit® for the three urban settings which takes three steps to be ready for the simulation, starting with the building model, then converting it to a parametric model that changes according to the required variables, and ending by categorizing each element within the model to its proper layer and floor type to be ready for simulation.

#### Setting up the base case model

**Fig. 11:**
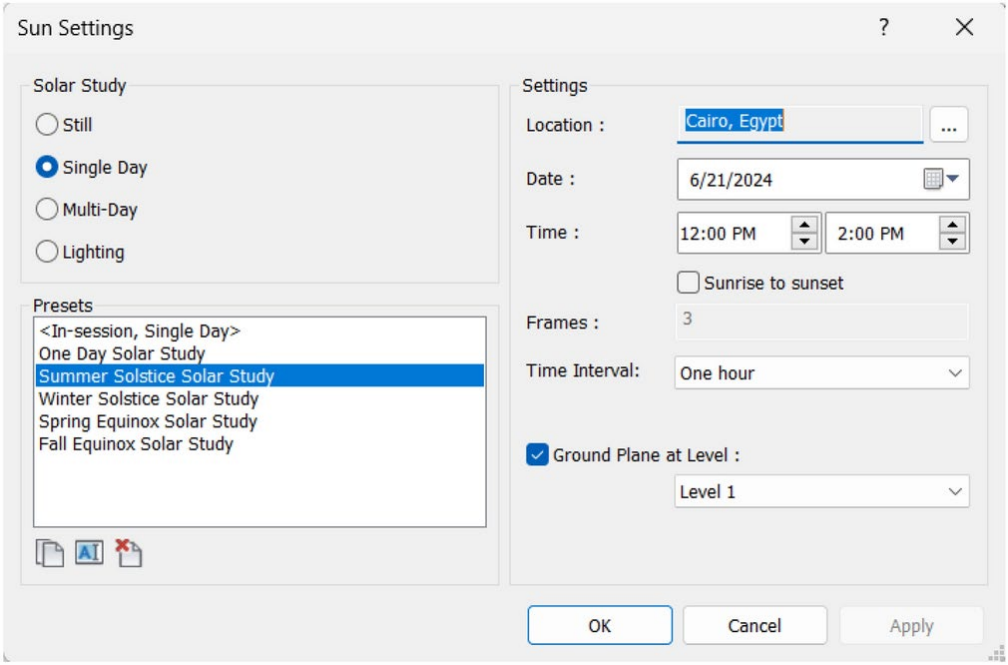
Sun Setting by the Author.

**Fig. 12:**
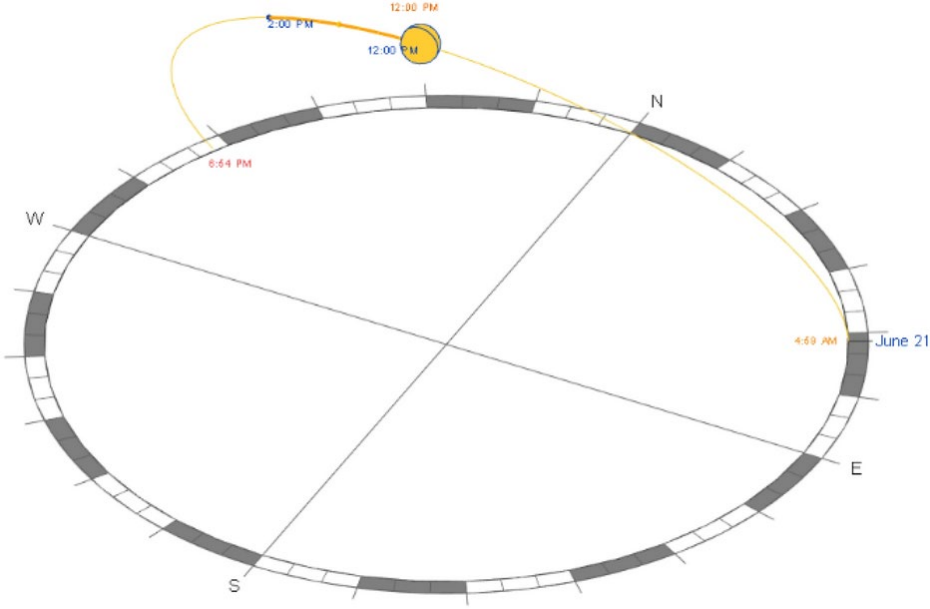
Sun Path and Time intervals inside the Revit model.

To ensure the accuracy of the measurement and setup process, all three neighborhoods were modeled with their real size and measurements. In addition, the sun setting is modified to be on the 21^st^ of June which is the longest day of the year with the most sunlight. To provide the highest possible levels of solar radiation and sun exposure during the peak summer, accordingly this day is ideal for simulating extremely hot temperatures. Furthermore, for each of the three cases, the time interval and duration were set at noon (12:00 PM – 2:00 PM), respectively, since this is when the sun is at its highest point and temperatures normally peak. Accordingly, the highest levels of thermal stress can be obtained by simulating this period. (Figs. 11, 12).

**Fig. 13:**
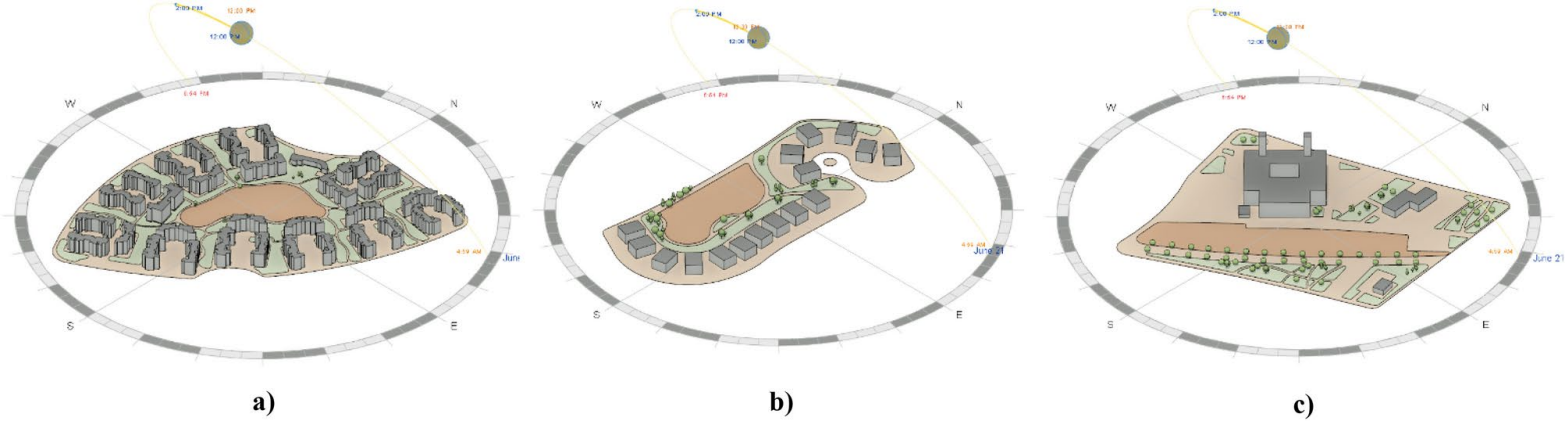
Revit Models for the three urban neighborhoods showing the solar path and sun setting by the Author. **a** Fully clustered area. **b** Semi-clustered area. **c** Open area.

In addition to the above considerations, the base case scenarios of the three urban settings were meticulously modeled, incorporating the precise building footprints and current distribution of vegetation. Additionally, all the component’s classifications are properly layered within the Revit model to ensure the integrity and reliability of the simulation and the results. (Fig. 13).

#### The algorithmic script for optimization

**Fig. 14:**
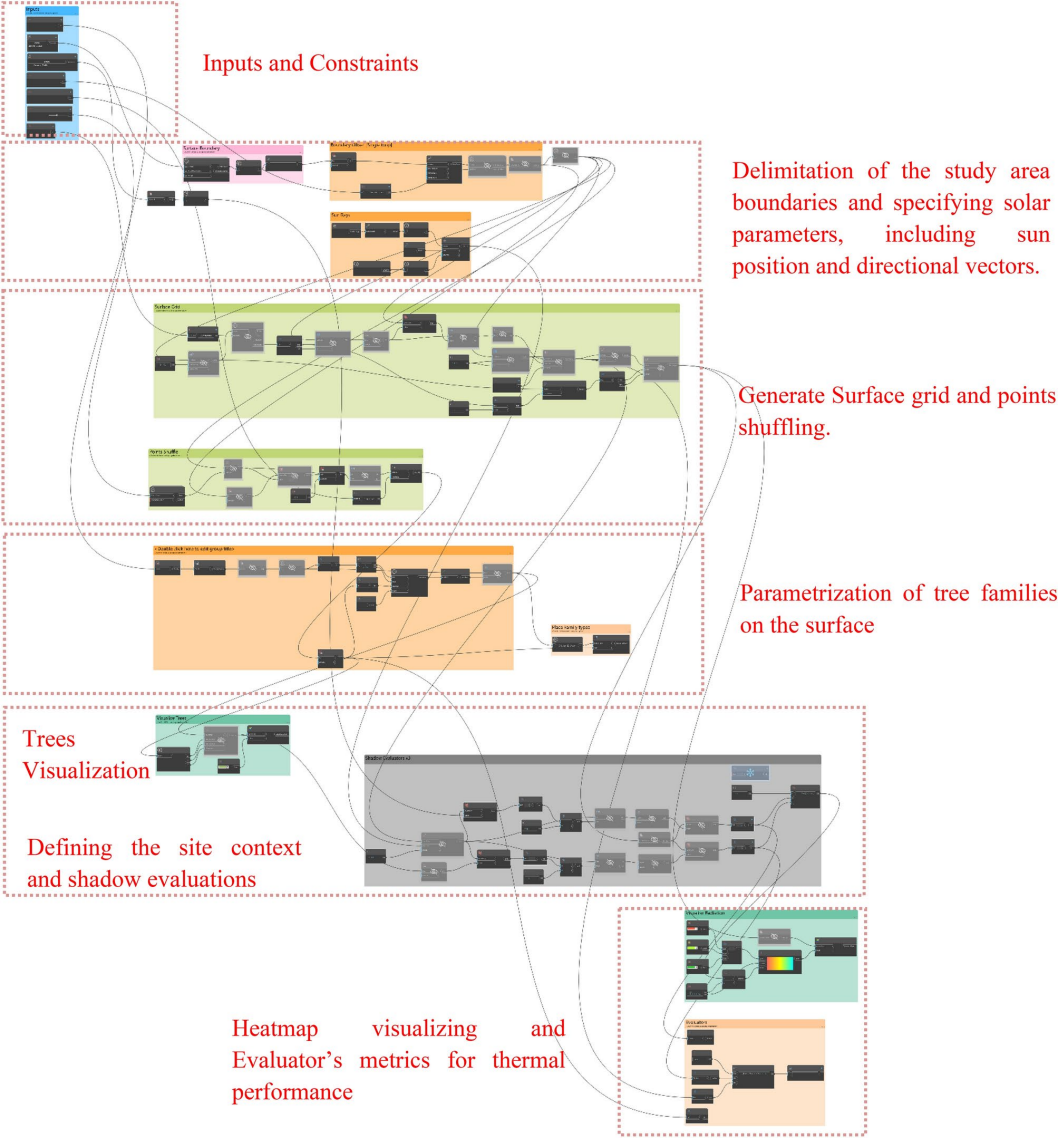
Dynamo script developed by the author.

This section discusses the algorithmic script of the Dynamo generative design tool, which was scripted to optimize the tree distribution within the three selected communal spaces. (Fig. 14).

**Fig. 15:**
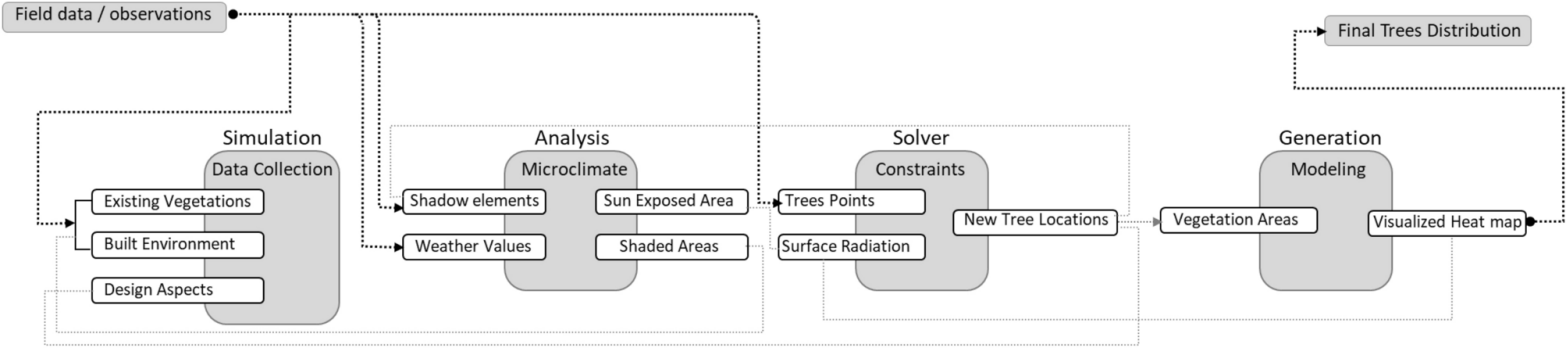
Inputs and outputs logic by the author.

Furthermore, the Dynamo script is divided into four main sectors and a set of interconnected components. To provide accurate output results, each component or node requires specific input parameters and functions, which can be optional or pre-defined. Additionally, each component in the Dynamo script generates output data, which are then fed into other components to create a chain and flow of information that eventually achieves the desired outcomes (Fig. 15). The following section provides an in-depth analysis and breakdown of each phase in the script logic design.

#### Input parameters and constraints definition

**Fig. 16:**
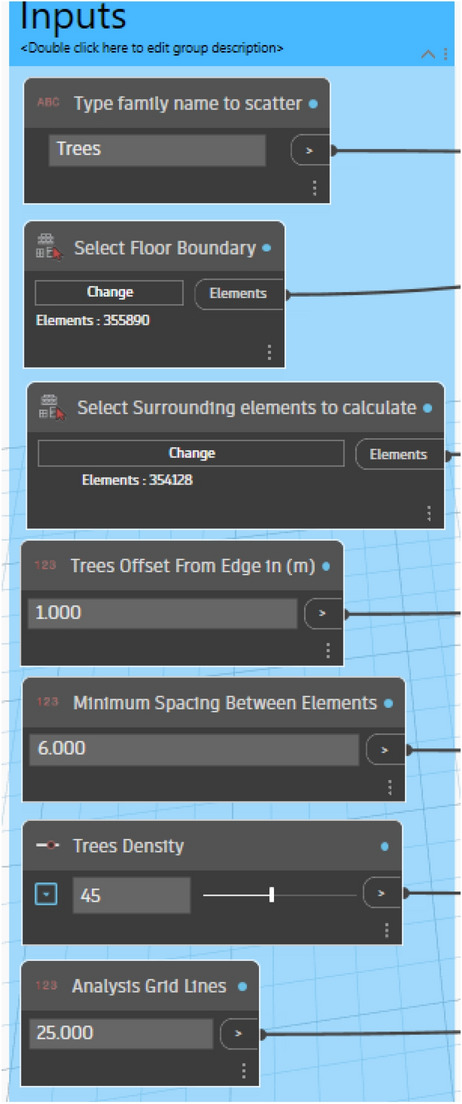
Inputs script design.

The initial phase involves determining the fundamental parameters and constraints required for the optimization process. This includes choosing the tree family to be distributed over the surface, through defining the surface boundary and identifying surrounding elements to be taken into account in the evolution process. Moreover, the tree offset, is designed to keep the tree 1 meter away from the surface edge, as this is considered an important constraint to ensure that trees are not positioned on the edge of the site boundary. Additionally, to avoid crowding and ensure healthy tree growth, the minimum distance between trees is set at 6 meters. Furthermore, the tree density was set at 45 units. Lastly, a finer grid will result in sharper and more accurate heatmaps; higher values indicate better precision in the visualization but a longer simulation time. Accordingly, the analytical grid lines for display are set at 25 out of 100. (Fig. 16).

#### Study area delimitation and solar parameter specification

In this phase, the study area is defined using the *‘Surface.ByPatch’* node to define its boundaries precisely. The solar parameters are then configured with *‘SunSettings.Current’* to determine the sun’s position which is set already in Revit file, *‘SunSettings.SunDirection’* to set the direction of sunlight, and *‘Coordinated.ProjectRotation’* to adjust the project’s alignment with the sun’s path. This setup allows accurate analysis of how sunlight impacts the study area.

#### Surface grid generation and points shuffling

A structured grid is generated across the pre-defined surface and points are dispersed randomly inside it. The *‘Surface.PointAtParameter’* node defines the positions of these points by using two key parameters, U and V. *U* generally defines the horizontal direction of the surface, spanning from 0 to 1, whereas *V* defines the vertical direction in the same range, in order to ensure uniform coverage of the entire surface, Meanwhile, the *‘Generate Random Points’* node is essentially randomizing the arrangement of the points, which is crucial for identifying the best spots to place trees, especially in regions that do not have much shade or where shadows are quite minimal.

**Fig. 17:**
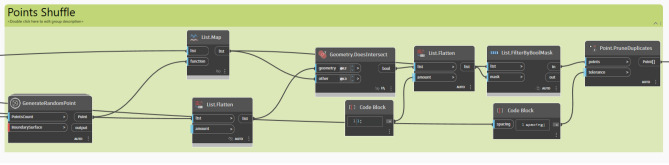
Points Shuffling across the surface script logic.

Additionally, to ensure that there are no duplicated points, and that each generated point is single and distinct, *‘Points.PruneDuplicate’* node is used to eliminate any duplication. Furthermore, the *‘Geometry.DoesIntersect’* node checks for any overlap between the generated points and any existing elements in the study area, whereas the *‘List.FilterByBoolMask’* node filters out and excludes points that do not meet the abovementioned criteria, ensuring that only pertinent points are used for the analysis and optimization process. (Fig. 17).

#### Parametrization of tree families on the surface

**Fig. 18:**
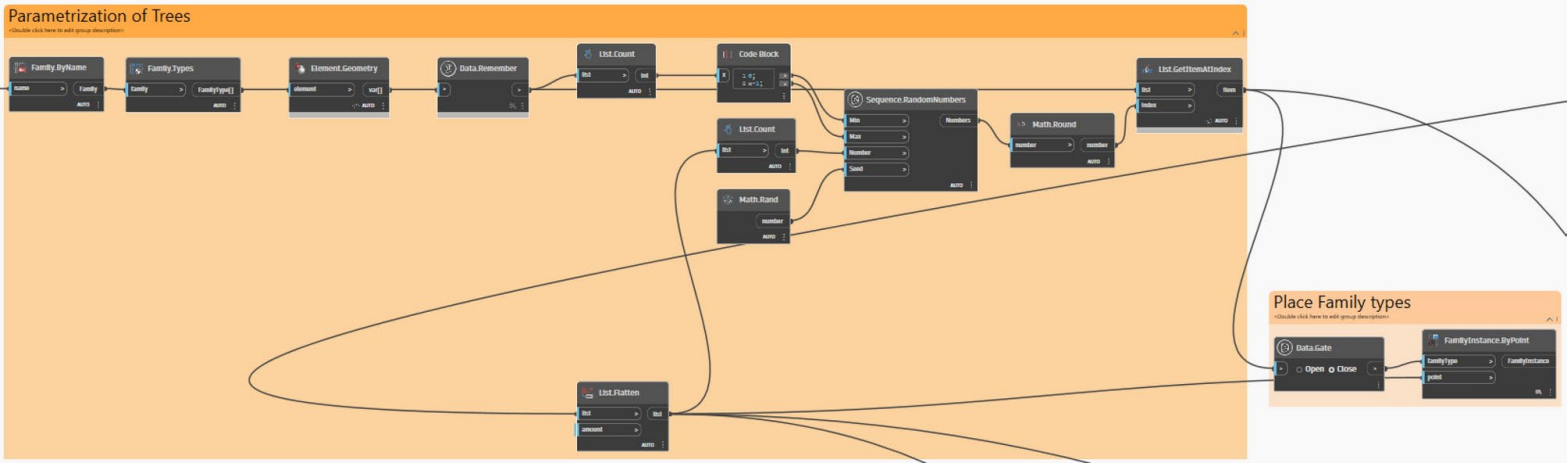
Trees placing sequential logic.

In this phase, the script logic is to parametrize tree families to be positioned on the pre-generated grid points in the previous phase. Moreover, the procedure of this phase starts with the *‘Sequence.RandomNumbers’* node, which generates a list of randomized numbers within a specified range and is coded as follows: 0 (minimum value) to X- 1 (maximum value), where X represents the total number of grid points. These random numbers can be used as potential indices for choosing the grid points where trees will be distributed and placed accordingly. However, because the numbers generated might contain decimal values, the *‘Math. Round’* node is used to round these numbers to the nearest whole numbers, ensuring that they are valid indices. Finally, the *‘FamilyInstance.ByPoint’* node places tree instances at these chosen points, ensuring a proper distribution across the surface and no trees are placed outside the boundaries. (Fig. 18).

#### Visualization of tree placement

**Fig. 19:**
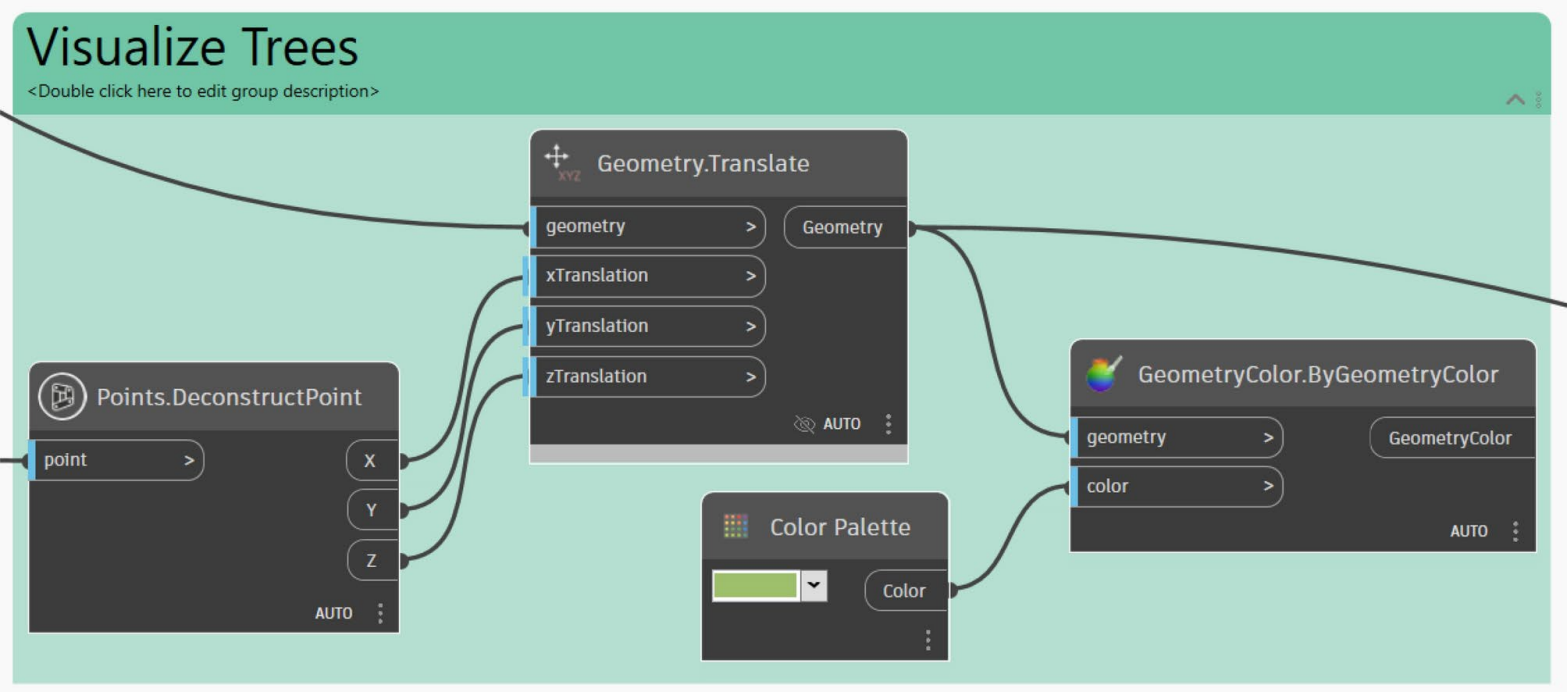
Trees visualizing logic.

The *‘Points.DeconstructPoint’* node, is used which divides point coordinates into X, Y, and Z components, allowing visualization of the placed trees. The *‘Geometry.Translate’* node is then used to convert these coordinates into 3D geometry, enabling the spatial representation of the tree placements. Furthermore, to enhance visual clarity, the *‘Geometry.Color’* node is applied to color the trees in green, to distinguish them visually from other elements. (Fig. 19).

#### Site context and shadow evaluation

**Fig. 20:**
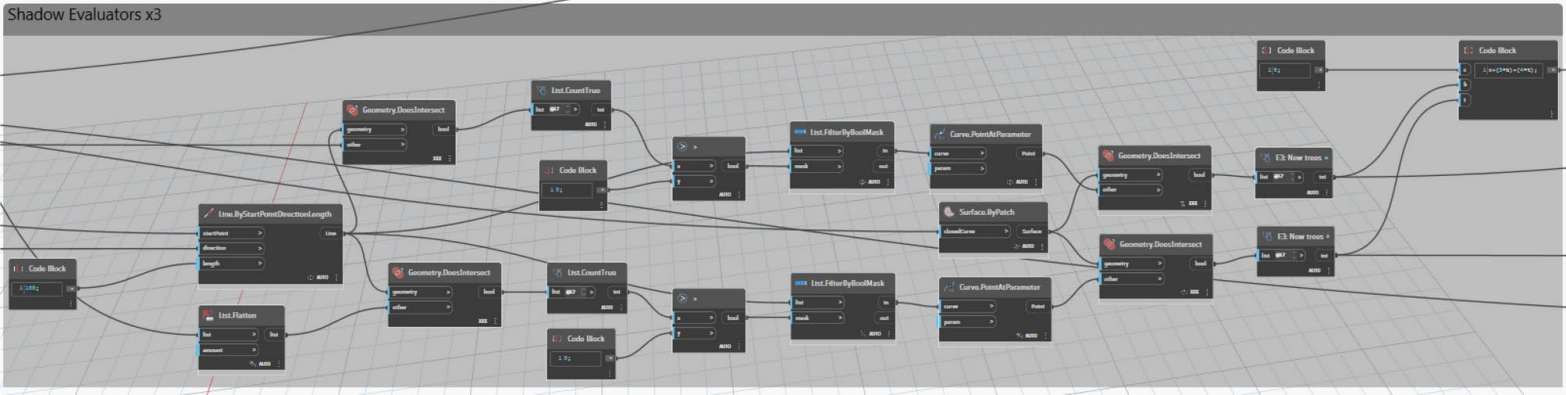
Shadows evaluators logic.

This phase is mainly for evaluating shadow impacts on the study area. By using the *‘Shadow.BySunPosition’* node which is mainly used to simulate and analyze shadows cast by surrounding existing elements as well as newly placed trees. (Fig. 20).

#### Heatmap visualization

A visual representation of heatmaps is used to evaluate the different amounts of solar exposure on the surface. The *‘Surface.ByPatch’* node is used to specify the locations for which the solar exposure will be evaluated and measured. In Addition to that, the *‘Geometry.ByColor’* node uses these exposure data to represent a color-coded scheme over the study surface area, where the green color represents minimal exposure (value of 0), the orange color indicates medium exposure (value of 0.5), and the red color signifies high exposure (value of 1).

#### Final evaluation and coverage calculation

**Fig. 21:**
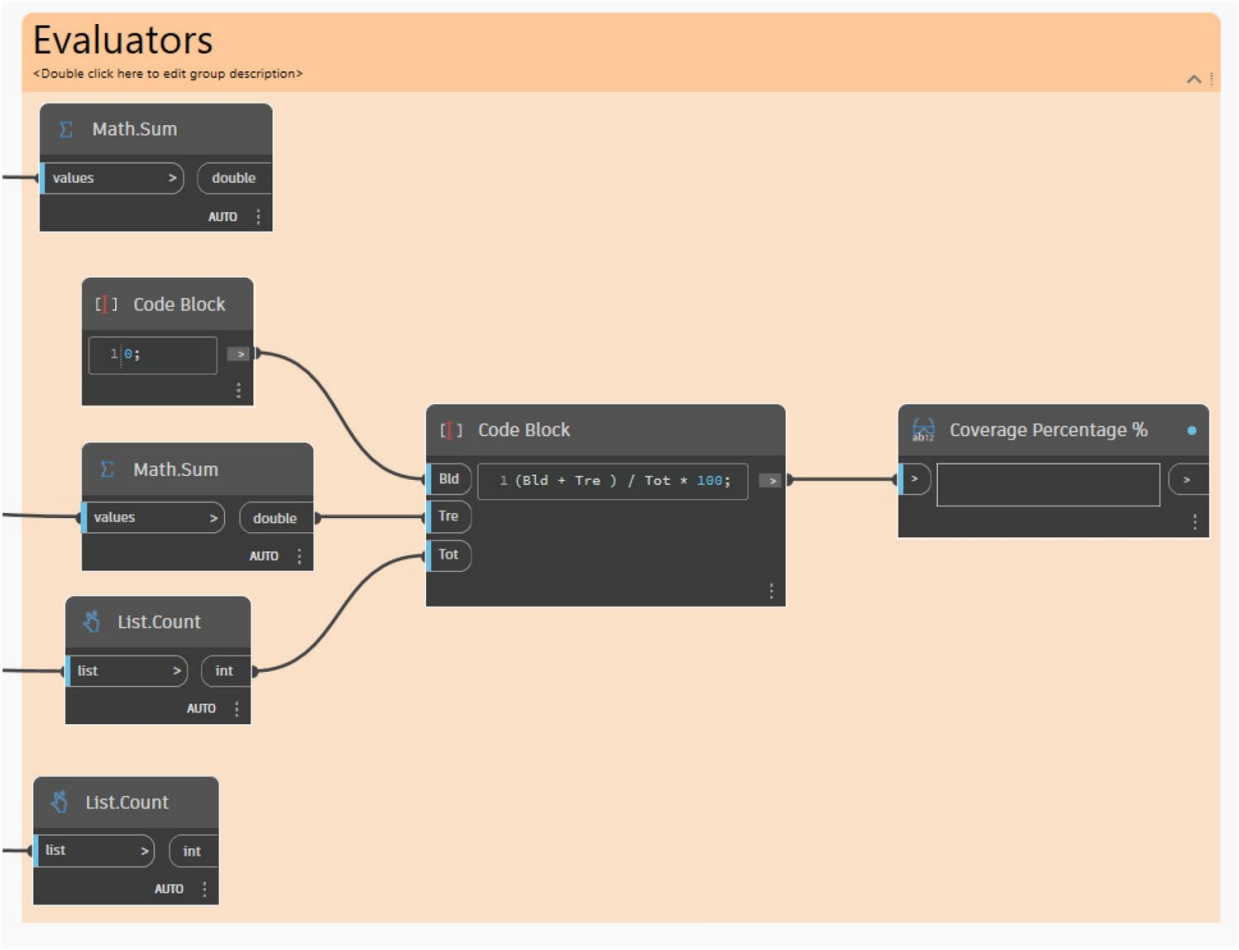
Evaluator/Outputs.

The final phase primarily evaluates the efficacy of the tree distribution by calculating and determining the coverage percentage on the surface. The formula used to calculate the percentage is **(Buildings + Trees/Total) × 100**. Note that, the building’s value is set to 0 since no buildings are present on the modeled surface. Accordingly, this percentage indicates how much of the surface is covered by trees, which helps to assess the effectiveness of the optimization process and ensures that the tree placement design meets the required environmental objectives. (Fig. 21).

### Generative design optimization process

The main intent of this section is to use the generative design tool to find the best possible design scenarios for tree placements that could improve outdoor thermal comfort in the three selected study cases. There are two primary methods are used to optimize the design which are:

1. **Randomization method:** This method shows a broad spectrum of design options and solutions by randomly varying input parameters, which provides an advantage to designers who are manually reviewing and filtering scenarios based on specific preferences or criteria.
2. **Optimization method:** While this method focuses on identifying the best solution through an iterative design refinement process based on predetermined criteria; however, it does not provide an opportunity for designers to filter out or choose from other potential alternatives. Basically, this approach provides the optimum scenario in terms of the tree coverage percentage while neglecting the count number of trees used.

Therefore, the randomization method will be selected for this research due to its ability to ease the manual evaluation and selection of the optimal scenario that achieves the highest coverage percentage while minimizing the number of trees (with the least tree count possible). Accordingly, this approach allows for an inclusive exploration of various design alternatives while ensuring that the final solution balances both coverage efficacy and tree count.

#### Simulation setup

**Fig. 22:**
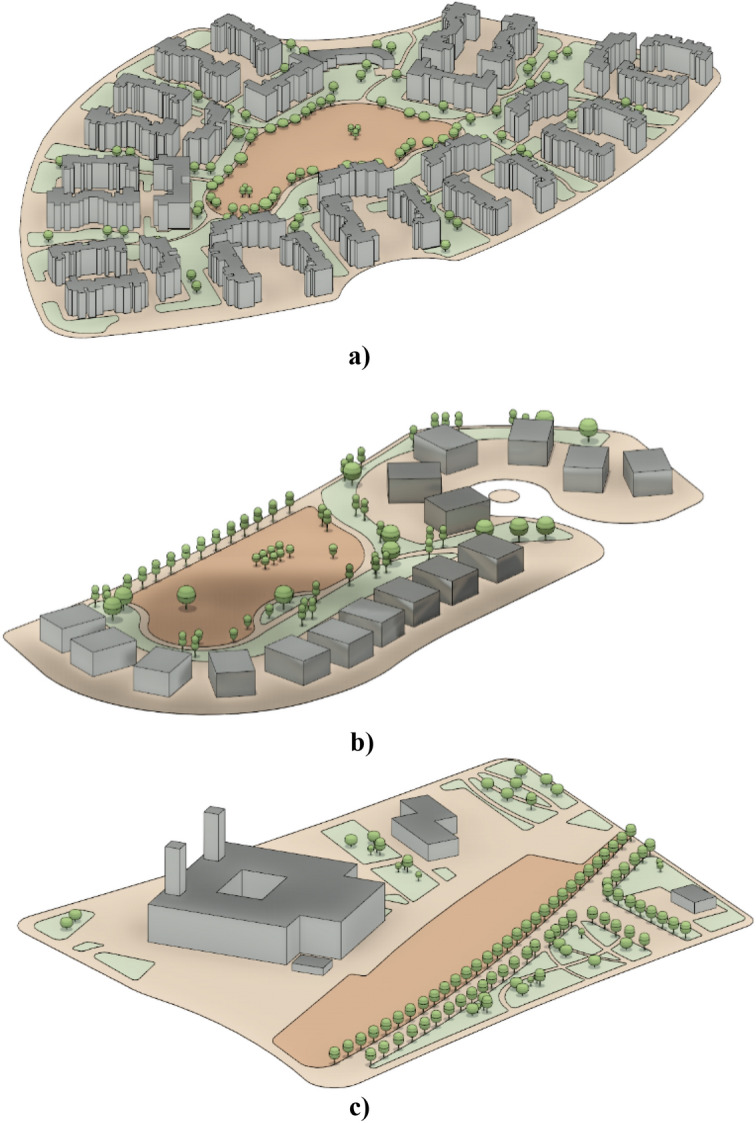
Base case scenarios with existing vegetation for the three urban neighborhoods by the Author. **a** Existing clustered enclosed neighborhood 3D model. **b** Existing semi-clustered neighborhood 3D model. **c** Existing fully open neighborhood 3D model.

**Fig. 23:**
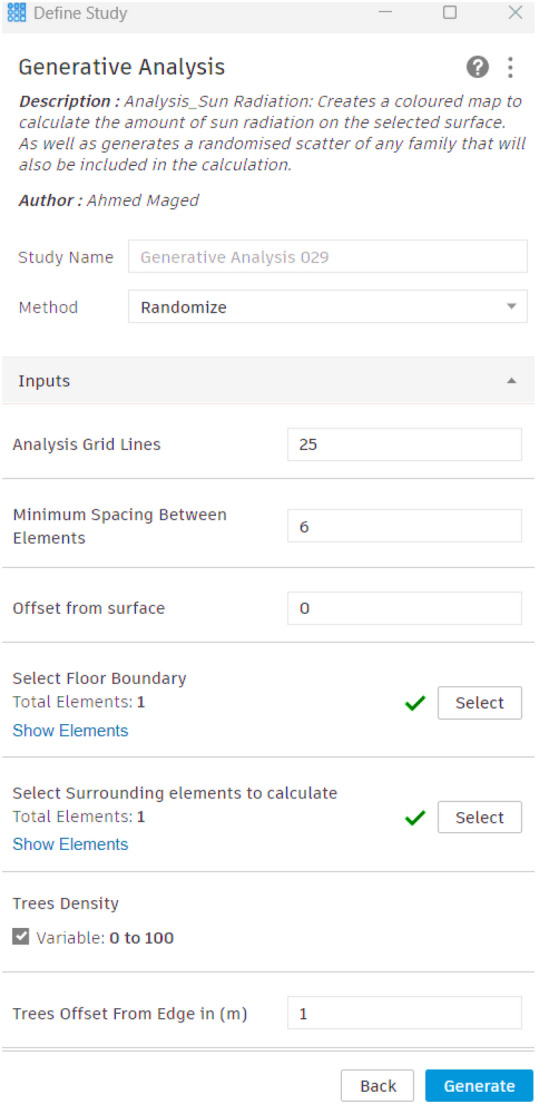
Generative tool study setup by the Author.

The model was created using actual tree locations and real measurements for every tree family to ensure that the scattered families correspond to the trees that are already located at the site and even had the same species selection (Figs. 22,23). Moreover, the input data were set as follows:

- **Analysis grid lines:** Set to 25 (more grid lines lead to longer simulation times).
- **The minimum spacing between trees:** 6 m to ensure there are no clashes between elements.
- **Offset from the surface:** 0 to scatter the tree family at the ground surface level.
- **Offset from the edge of the site:** 1 meter to avoid being on the exact edge of the site.
- **No. of solutions:** 200 simulations per each neighborhood
- **No. of seeds:** 5 seeds (which means that the tool will generate five different sets)

## Optimization results and solutions

The process begins by defining the input parameters as mentioned in the preceding section, through identifying the surface of each neighborhood, setting the minimum feasible spacing between trees, and determining the constraints related to surface offsets and edge distances. (Fig. 23) Accordingly, an iterative optimization technique was used with these parameters to methodically investigate different tree placement configurations. The main objective was to determine the arrangement that achieved the highest possible percentage of tree coverage with the minimal tree counts possible and the results are shown as follows:

### Clustered enclosed neighborhood area

**Fig. 24:**
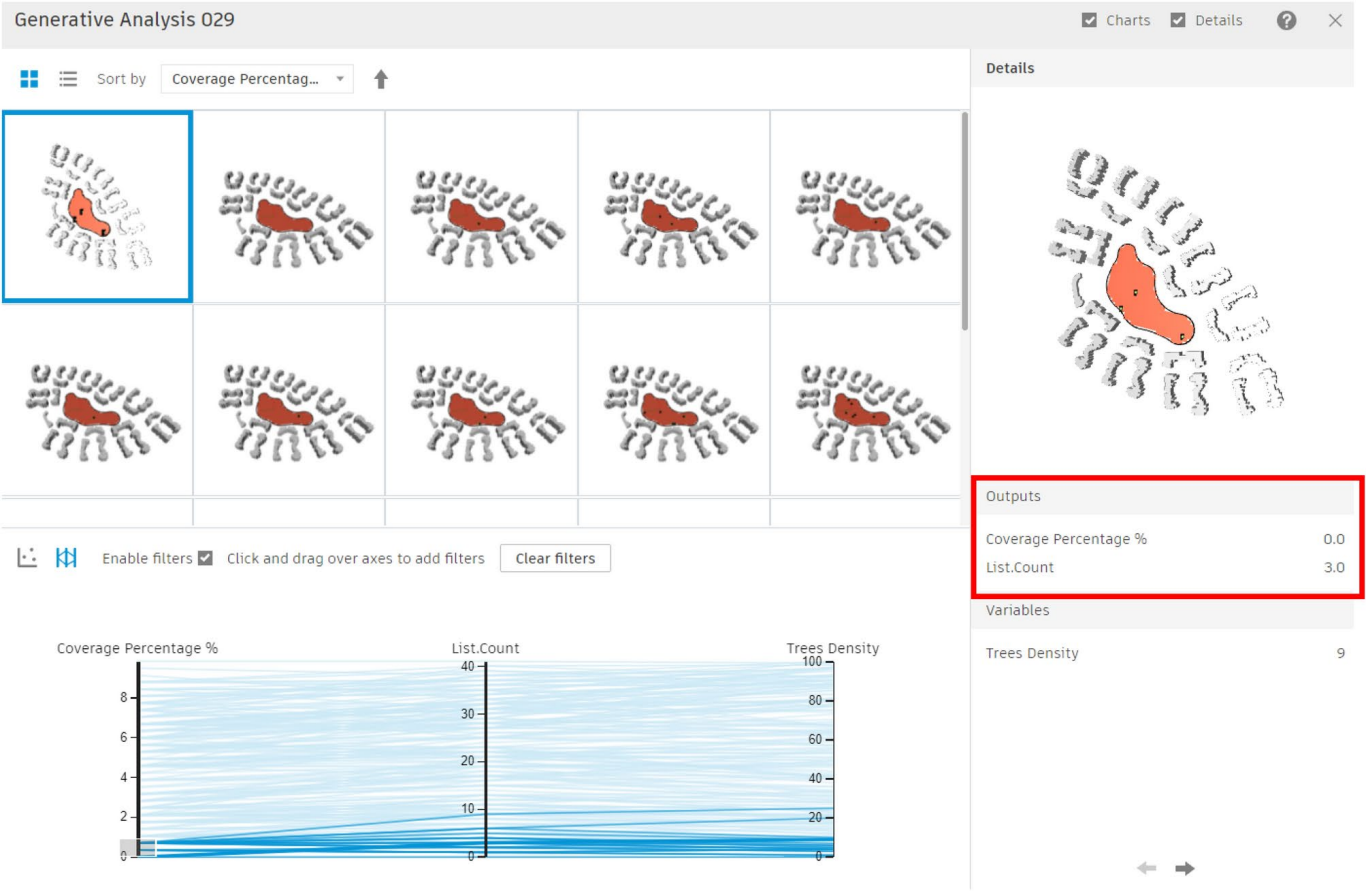
Generated design solutions with the least coverage percentage 0%.

**Fig. 25:**
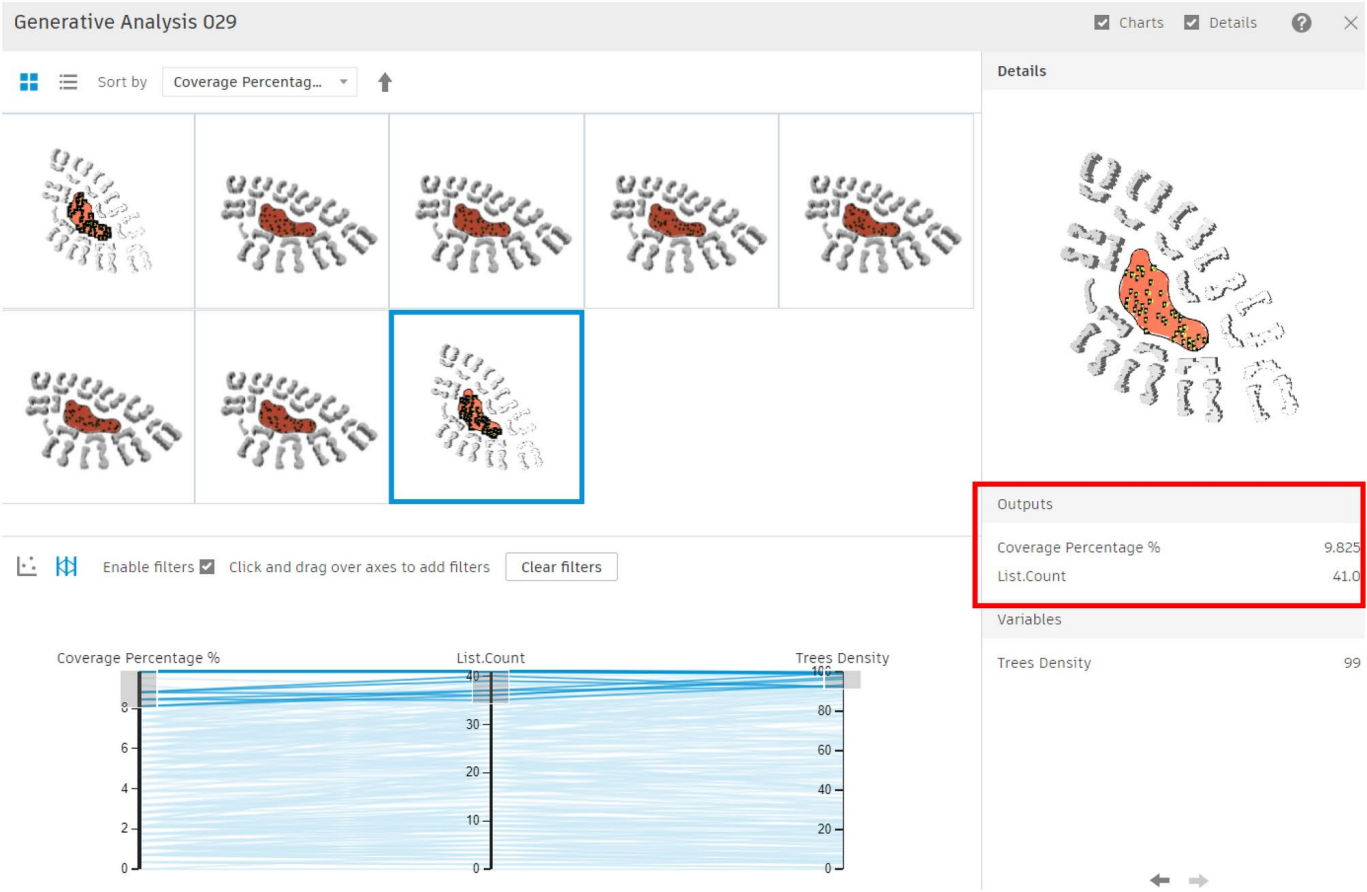
Generated design solutions with the Maximum coverage percentage 9.825%.

For this area, 200 simulations with 5 seeds were conducted, showing results that varied from a minimum coverage percentage of 0% with approximately 3 trees (Fig. 24) to a maximum coverage of 9.82% with nearly 41 trees (Fig. 25). Meanwhile, the filters were divided into three main sections: coverage percentage, in relation to tree count, and tree density.

**Fig. 26:**
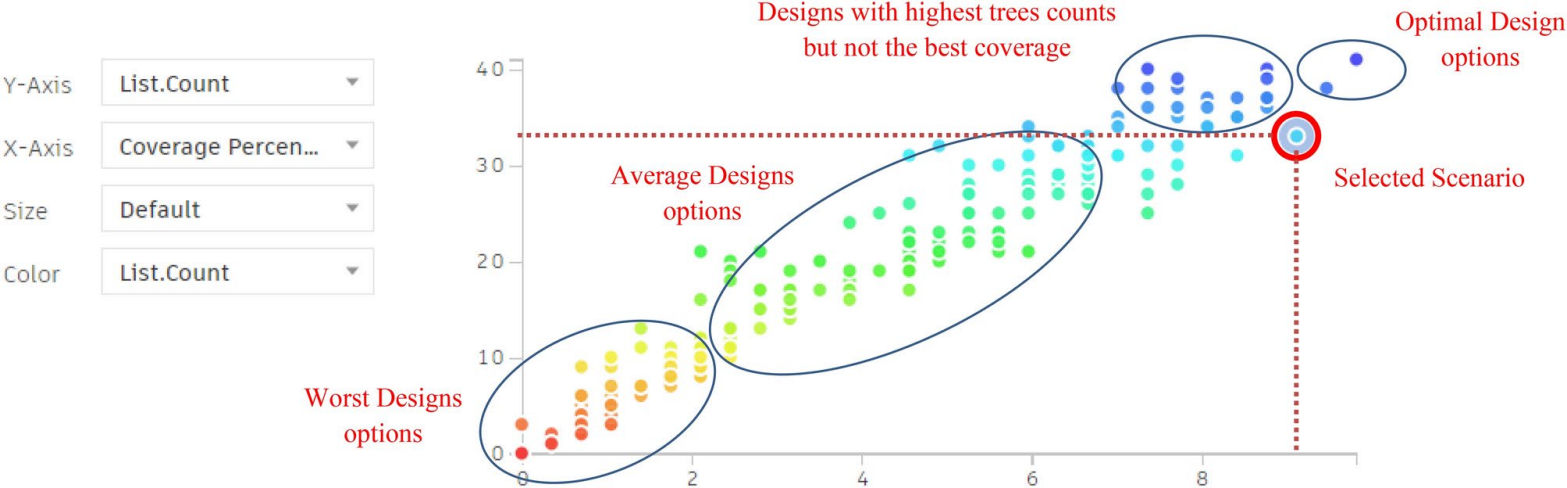
The graphical representation of the optimization results (Clustered neighborhood).

 The chart below shows a direct correlation between coverage percentage and tree counts (Fig. 26), which provides insights into how the generative design tool operates. This tool methodically investigates and optimizes solutions by using the logic of seeds and population sizes, rather than producing random scenarios. Each seed initializes a unique sequence of random numbers, generating diverse sets of initial solutions. For example, with 5 seeds, the tool creates five separate sets of solutions, each set representing different starting points for the optimization process as presented in the equation below.

Additionally, within each seed, the tool evaluates an initial population of solutions. For example, the first seed generates 40 solutions as the baseline population size. These solutions are evaluated and refined in subsequent populations, progressively becoming more aligned with the optimization objectives. As the process advances through the second and third populations, the tool iterates on the initial solutions, applying evolutionary strategies to enhance their quality.

$$Population\: size= \frac{200 \:solution}{5 \:seeds}=40\: solution\: per \:each\: 1 \:seed$$

Accordingly, by increasing the number of seeds and simulation sets this directly will enhance the probability of obtaining more refined and optimum scenarios, but it may also increase the simulation time.

The chart below illustrates this process by correlating the coverage percentage with the tree count. The Y-axis represents the tree count, whereas the X-axis represents coverage percentage.

Moreover, the red dots presented on the chart below indicate solutions with the fewest tree counts, whereas the violet dots represent those with the highest tree counts. This visualization chart helps in selecting the most optimal case from the 200 simulated scenarios, highlighting the tool’s ability to generate both effective tree counts and high coverage percentages (Fig. 26).

#### The chart below shows different categories of results:

The red and orange dots represent the worst generated designs, which have the least coverage percentages and the fewest tree counts. The green and cyan dots in between indicate average solutions, with coverage percentages ranging from 3% to 6% and using between 15 and 30 trees.

While some other designs present in violet dots have a large number of trees, up to 40, however, their coverage percentage is not the highest, reaching only 8%.

The last two options at the end of the chart show the highest coverage percentages, approximately 9.5% and 9.4%, but with maximum tree counts of 41 and 39, respectively.

However, the red-circled option is the best one in terms of both sectors, giving a 9.1% coverage percentage and 33 trees, which is the fewest tree count among designs with over 9% coverage.

**Fig. 27:**
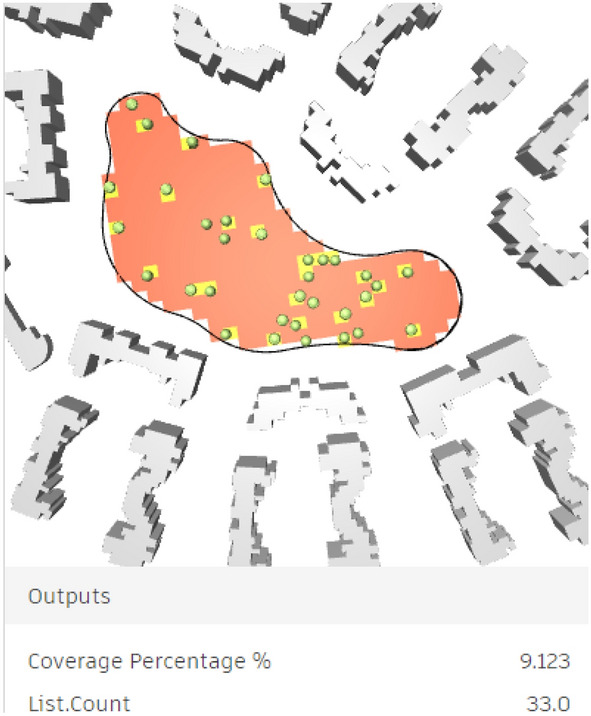
Selected scenario (Clustered neighborhood).

**Fig. 28:**
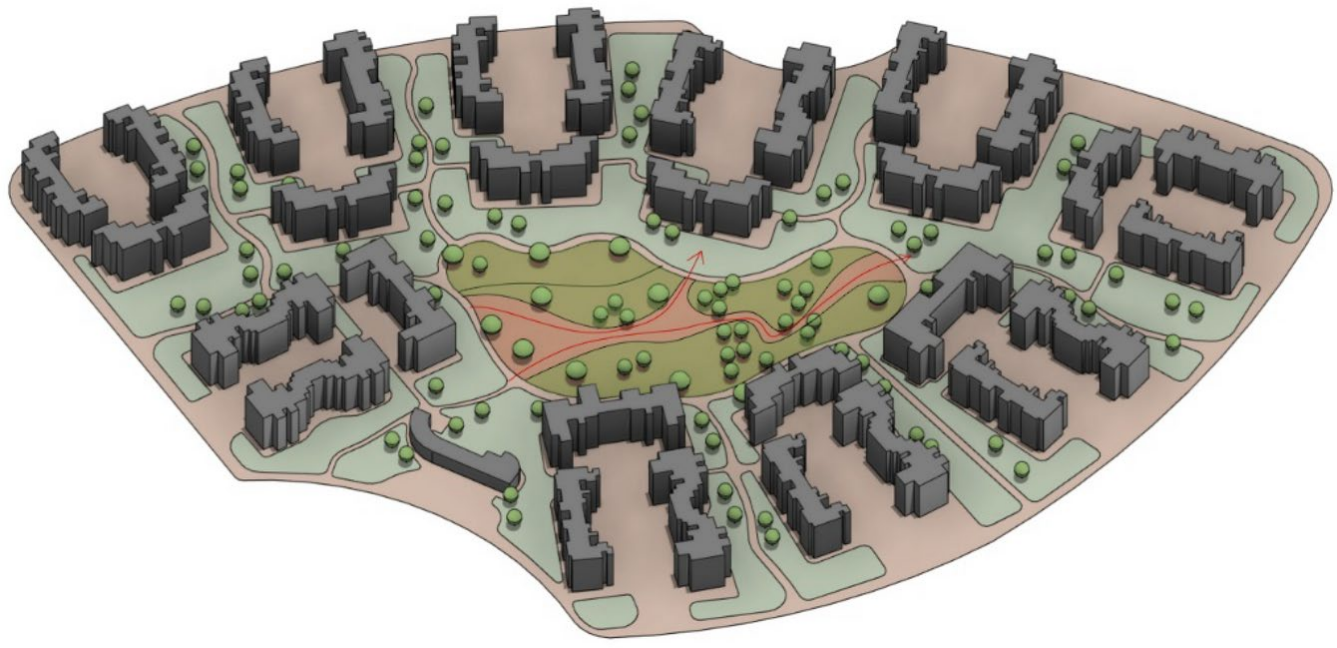
3D perspective shot with an abstractive design based on the new trees’ distribution with vegetation, patches, and walking pathways (Clustered neighborhood) by the Author.

As a result, by synchronizing the design elements with the model, the tree positioning was generally reasonable, and the spatial effects of the area were enriched (Figs. 27, 28). However, the environmental conditions and UTCI will be further examined for this scenario.

### Semi-clustered neighborhood area

**Fig. 29:**
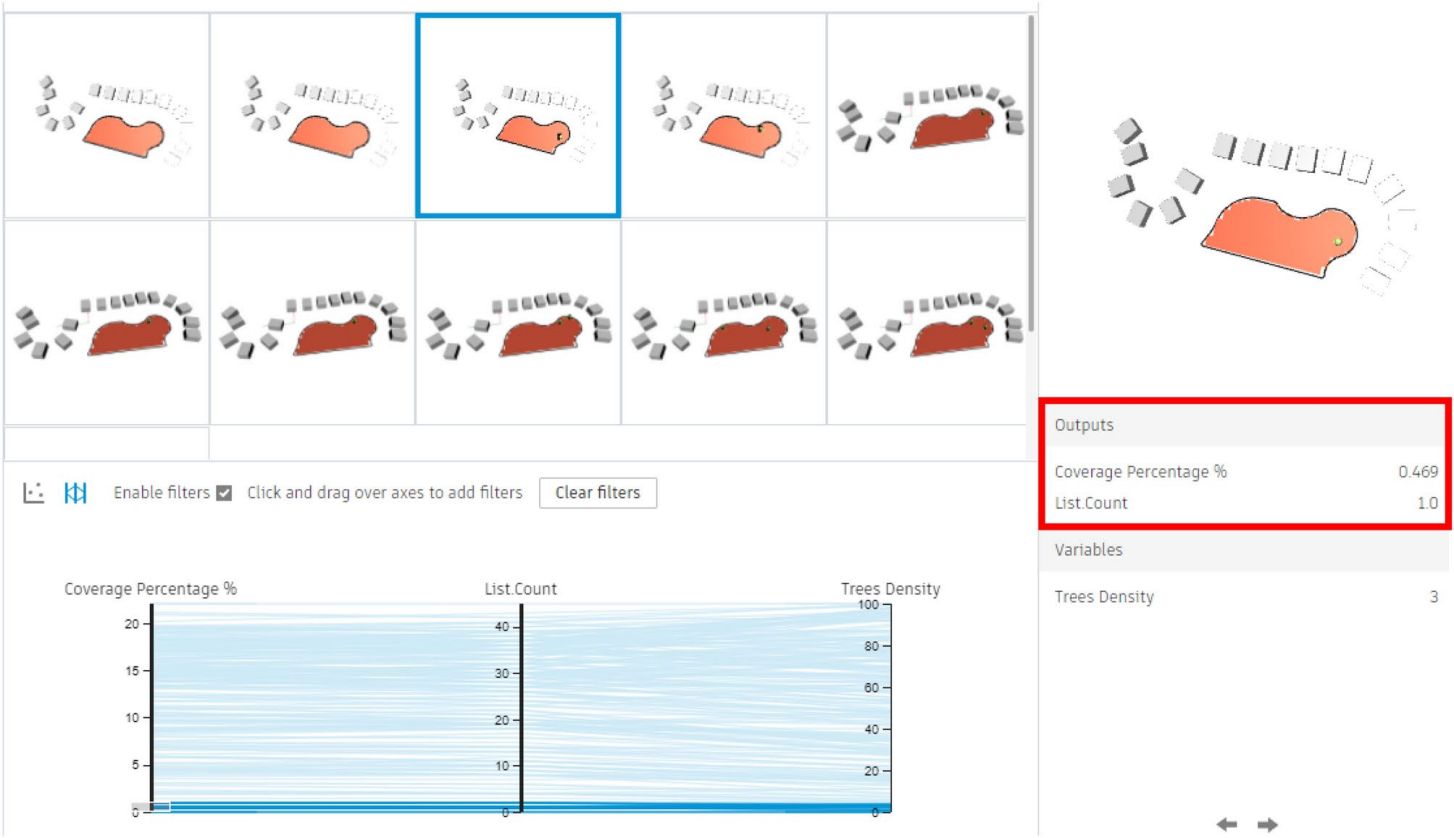
Generated design solutions with the least coverage percentage 0.46%.

**Fig. 30:**
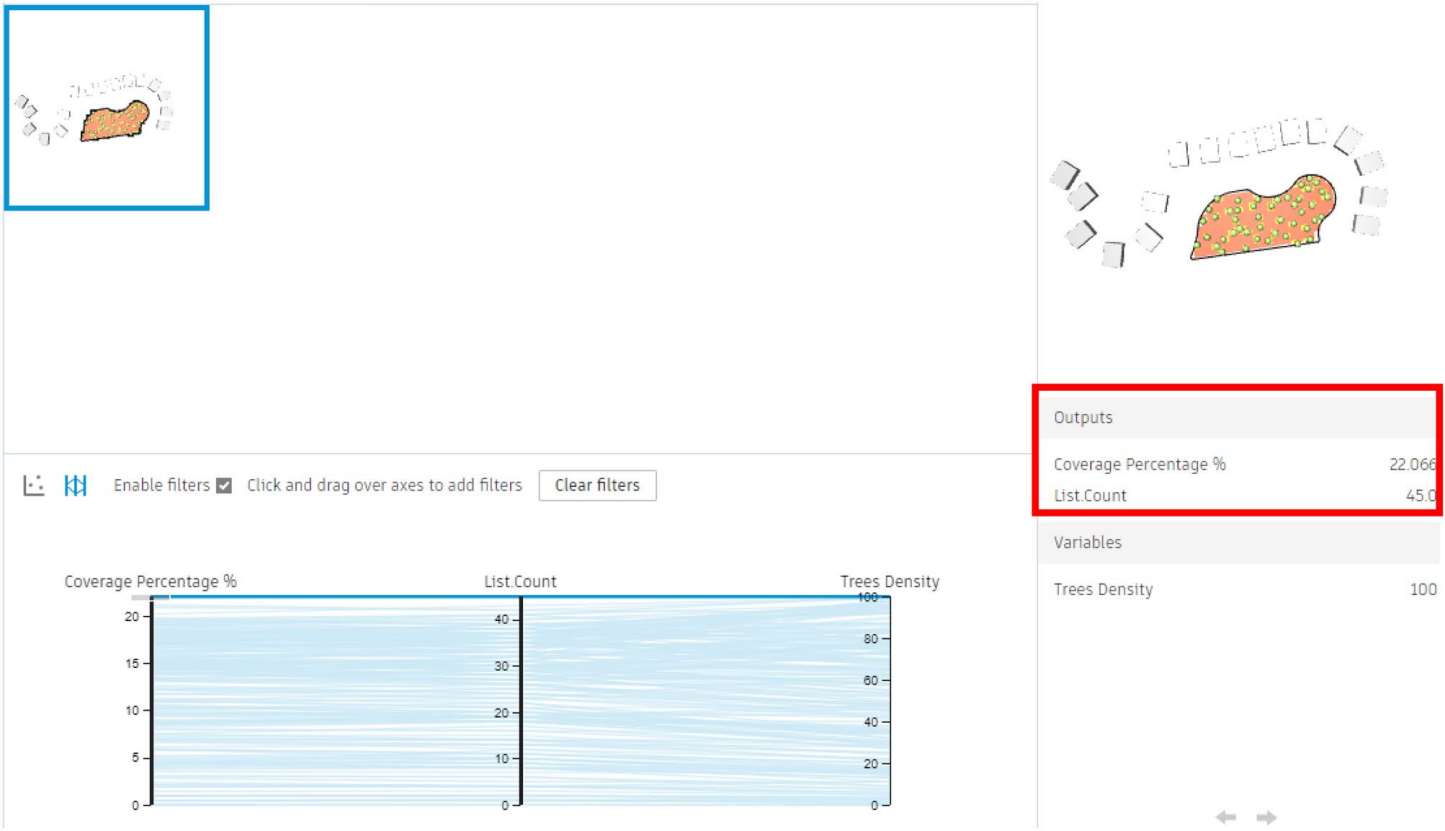
Generated design solutions with the Maximum coverage percentage 22.06%.

For the semi-clustered area, 200 simulations with 5 seeds were simulated, showing results varied from a minimum coverage percentage of 0.46% with only 1 tree (Fig. 29) to a maximum coverage of 22.06% with nearly 45 trees (Fig. 30).

#### Additionally, the chart below shows the following:

**Fig. 31:**
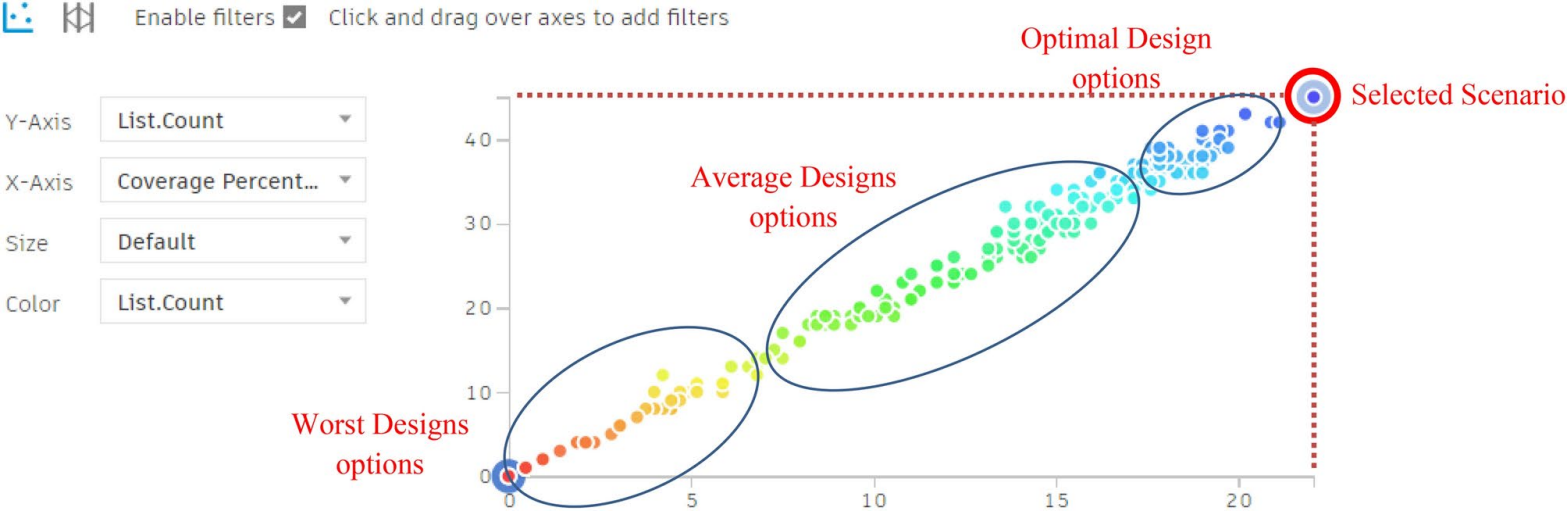
The graphical representation of the optimization results (Semi-clustered neighborhood).

As per the chart below (Fig. 31), the red and orange dots represent the worst design options, showing approximately 2% coverage with around 3–4 trees. While, the green and cyan dots indicate average options, providing almost 13% to 17% coverage with 30 to 35 trees.

The violet dots represent the best design solutions, with the highest coverage percentages, ranging from 20% to 22% with 40 to 45 trees.

However, the red-circled option is the best solution, offering 22.06% coverage with 45 trees.

**Fig. 32:**
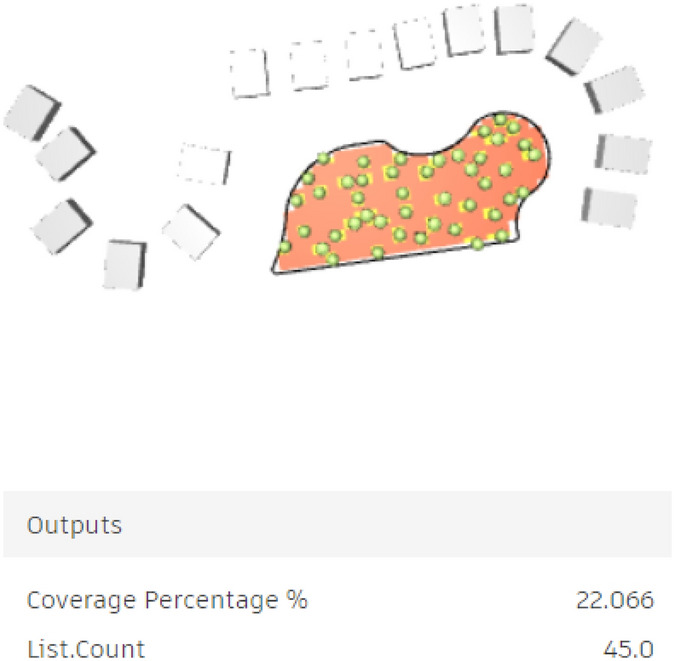
Selected scenario (Semi-clustered neighborhood).

**Fig. 33:**
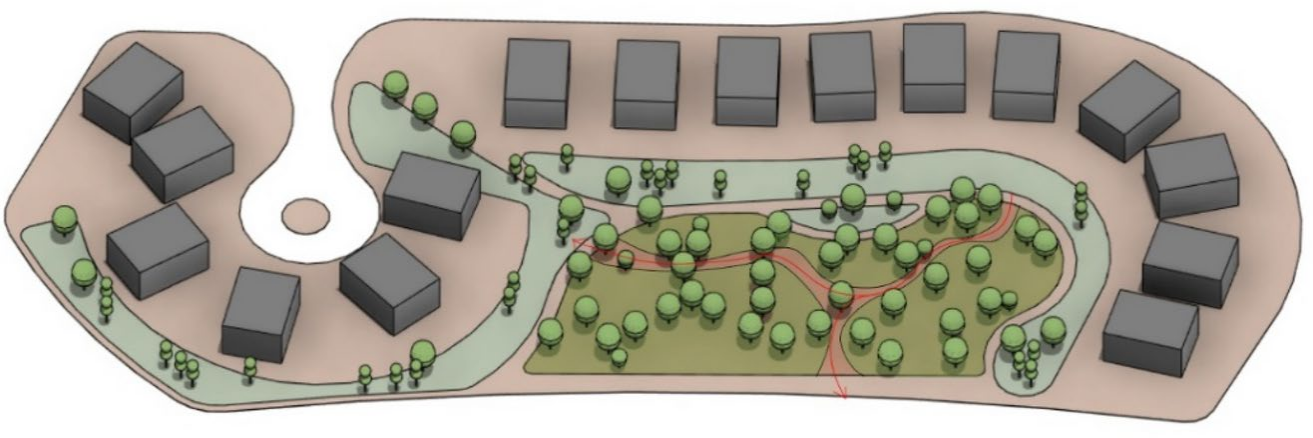
3D perspective shot with an abstractive design based on the new trees’ distribution with vegetation, patches, and walking pathways (Semi-clustered neighborhood) by the Author.

As a result, 45 trees were placed and synchronized in the model, forming 3 clusters of trees with an organic spine in between. However, the UTCI will be further examined for this case. (Figs. 32, 33).

### Fully open neighborhood area

**Fig. 34:**
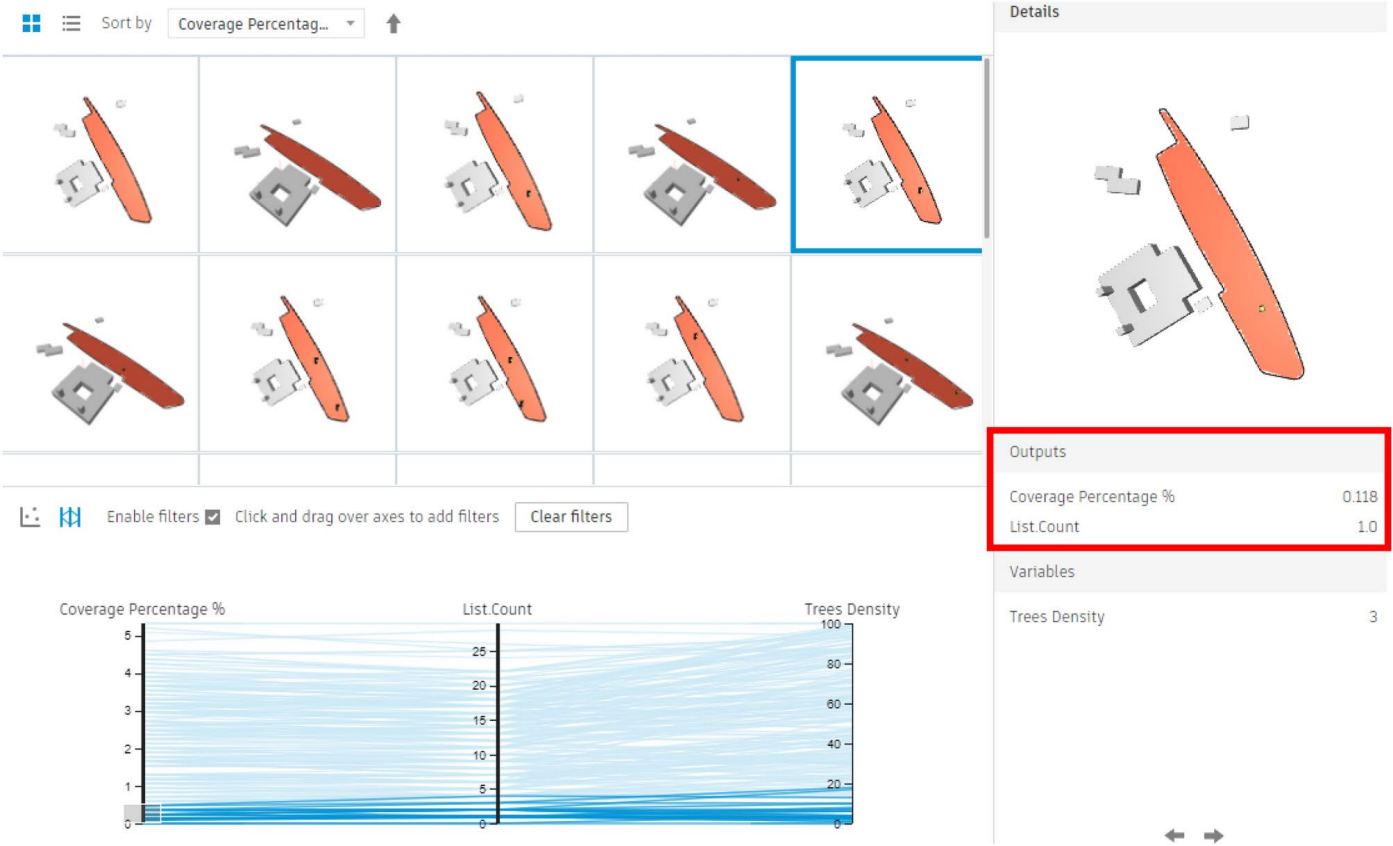
Generated design solutions with the least coverage percentage 0.11%.

**Fig. 35:**
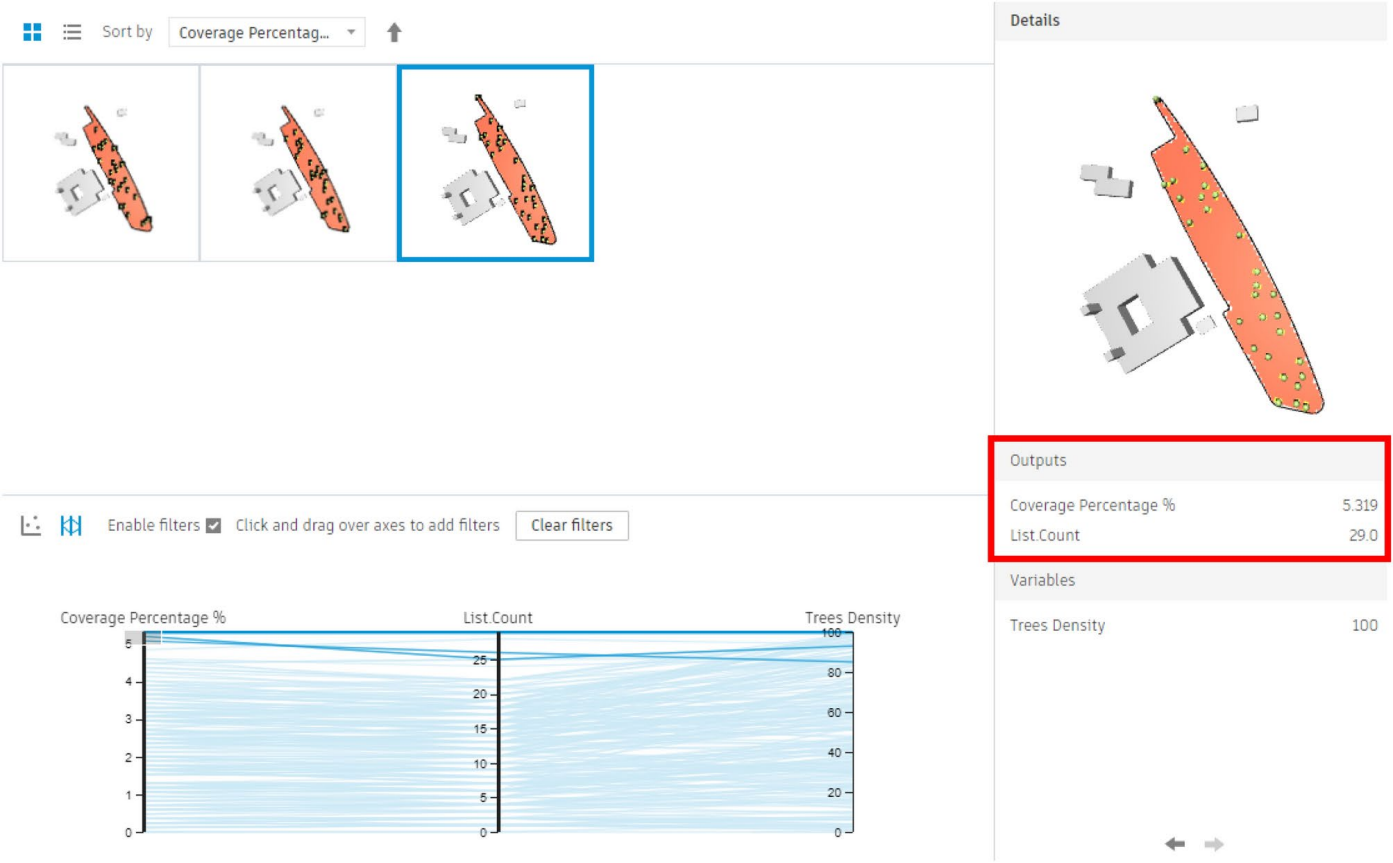
Generated design solutions with the Maximum coverage percentage 6.13%.

For the fully open area, 200 simulations with 5 seeds were conducted, showing results that varied from a minimum coverage percentage of 0.11% with only 1 Palm (Fig. 34) to a maximum coverage of 5.31% with only 29 palms (Fig. 35).

**Fig. 36:**
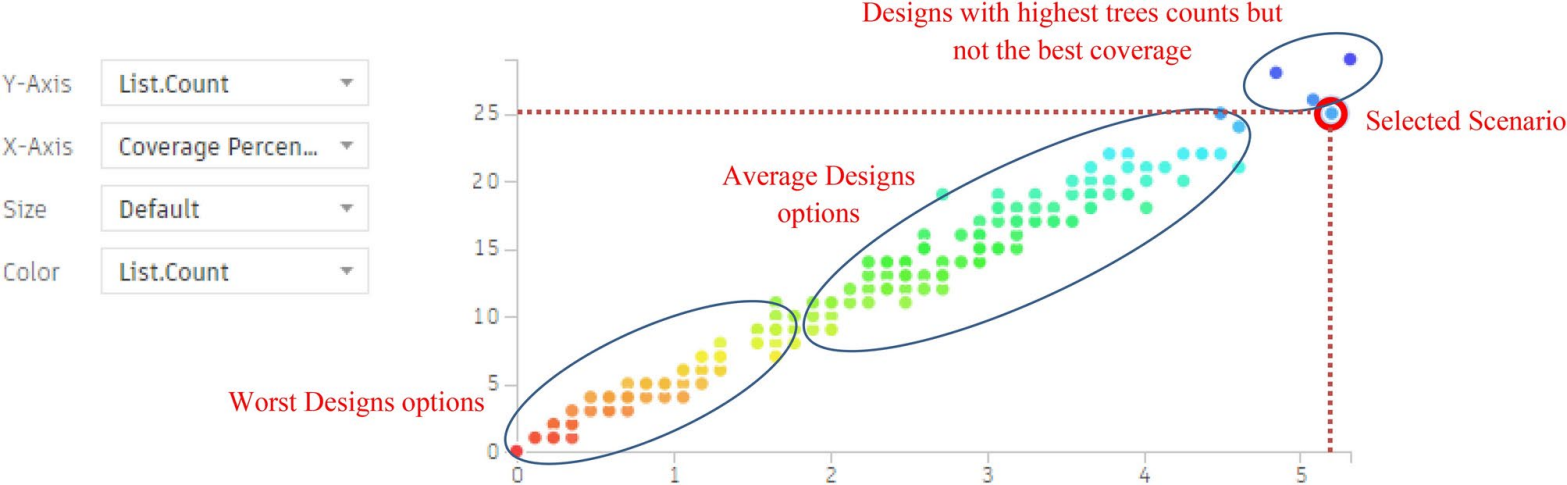
The graphical representation of the optimization results (Fully Open neighborhood).

Additionally, the chart below shows the following:

As per the chart below (Fig. 36), the red and orange dots represent the worst design options, showing coverage percentages ranging from 0.11% to 0.35% with around 2–3 palms. The green and cyan dots indicate average options, providing coverage between 2.12% and 4.37% with 12 to 22 palms.

The last three options at the end of the chart have the highest coverage percentages, approximately 4.84%, 5.08%, and 5.31%, but with maximum palm counts of 28, 26, and 29, respectively.

However, the red-circled option is the best scenario, offering 5.20% coverage with 25 palms, which is the fewest palm count among designs with over 5% coverage.

**Fig. 37:**
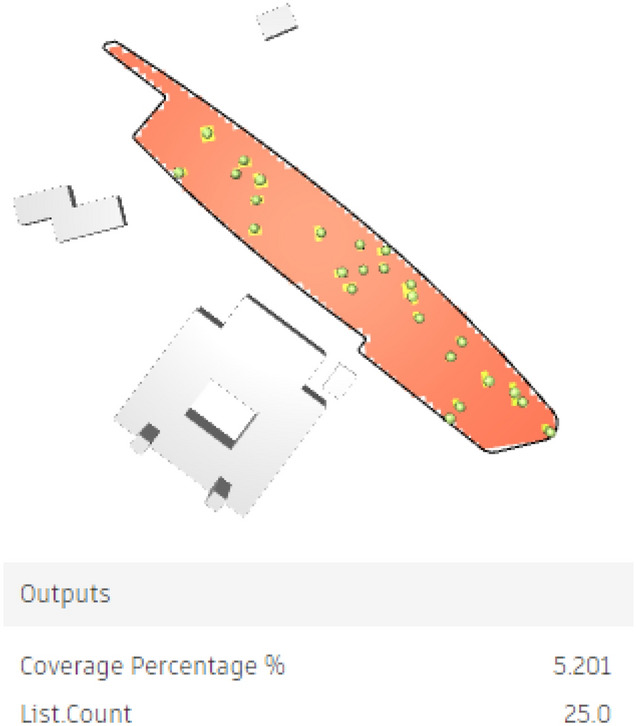
Selected scenario (Fully open neighborhood).

**Fig. 38:**
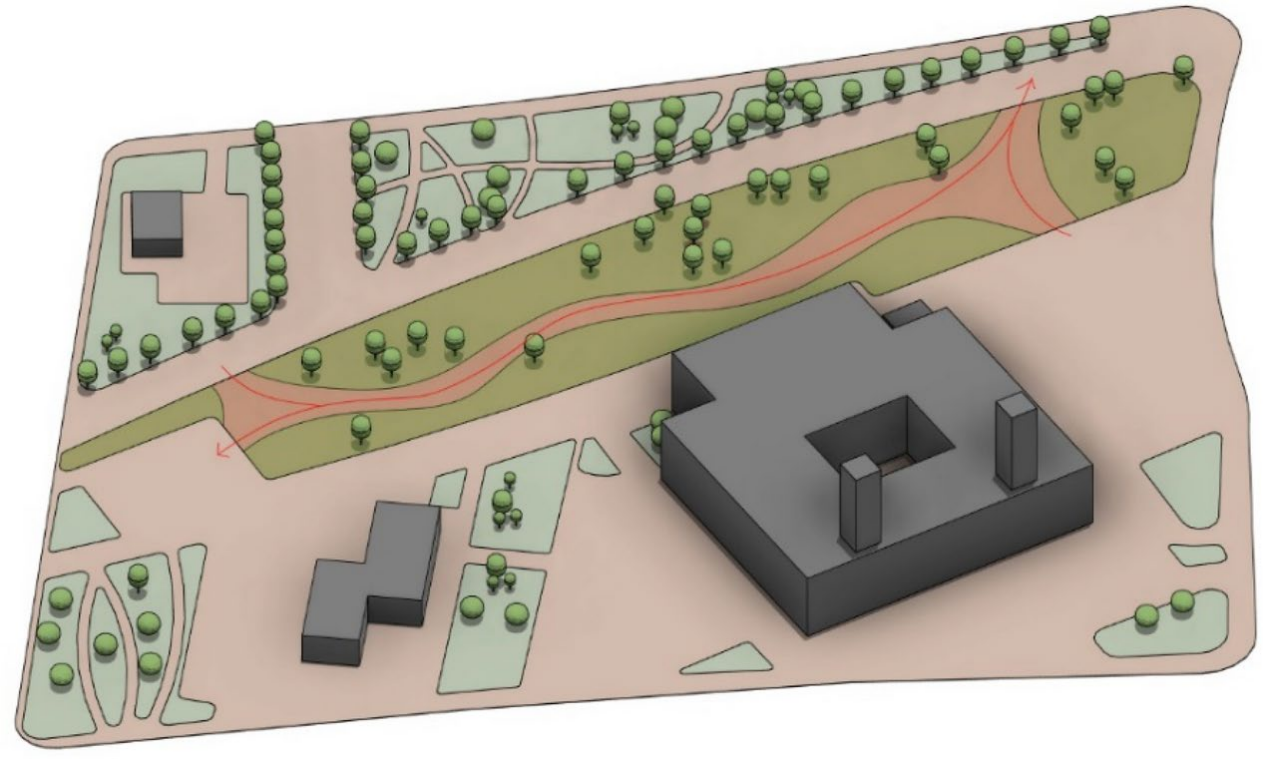
3D perspective shot with an abstractive design based on the new trees’ distribution with vegetation, patches, and walking pathways (fully open neighborhood) by the Author.

As a result, 25 palms were synchronized in the model, forming green beds with an organic spine in between them. However, UTCI will be further examined for this case. (Figs. 37, 38).

## Validation process

This phase is primarily for validating whether the generative design tool is considered an effective tool in the optimization process or not. Moreover, the validation procedures are divided into two phases: the first phase is to synchronize the Revit outputs with Rhino, and the second is to assess the UTCI values to evaluate the differences between the base cases and the optimized cases.

### Rhino.inside®Revit parametric design integration

The process starts by integrating the plugin into the Revit models for the 6 cases (3 base scenarios and 3 optimized scenarios) to streamline the exchange of geometries and data between both mentioned software platforms. In order to allow for a comparison in terms of UTCI and validate the effectiveness of the generative design tool in enhancing outdoor thermal comfort.

**Fig. 39:**
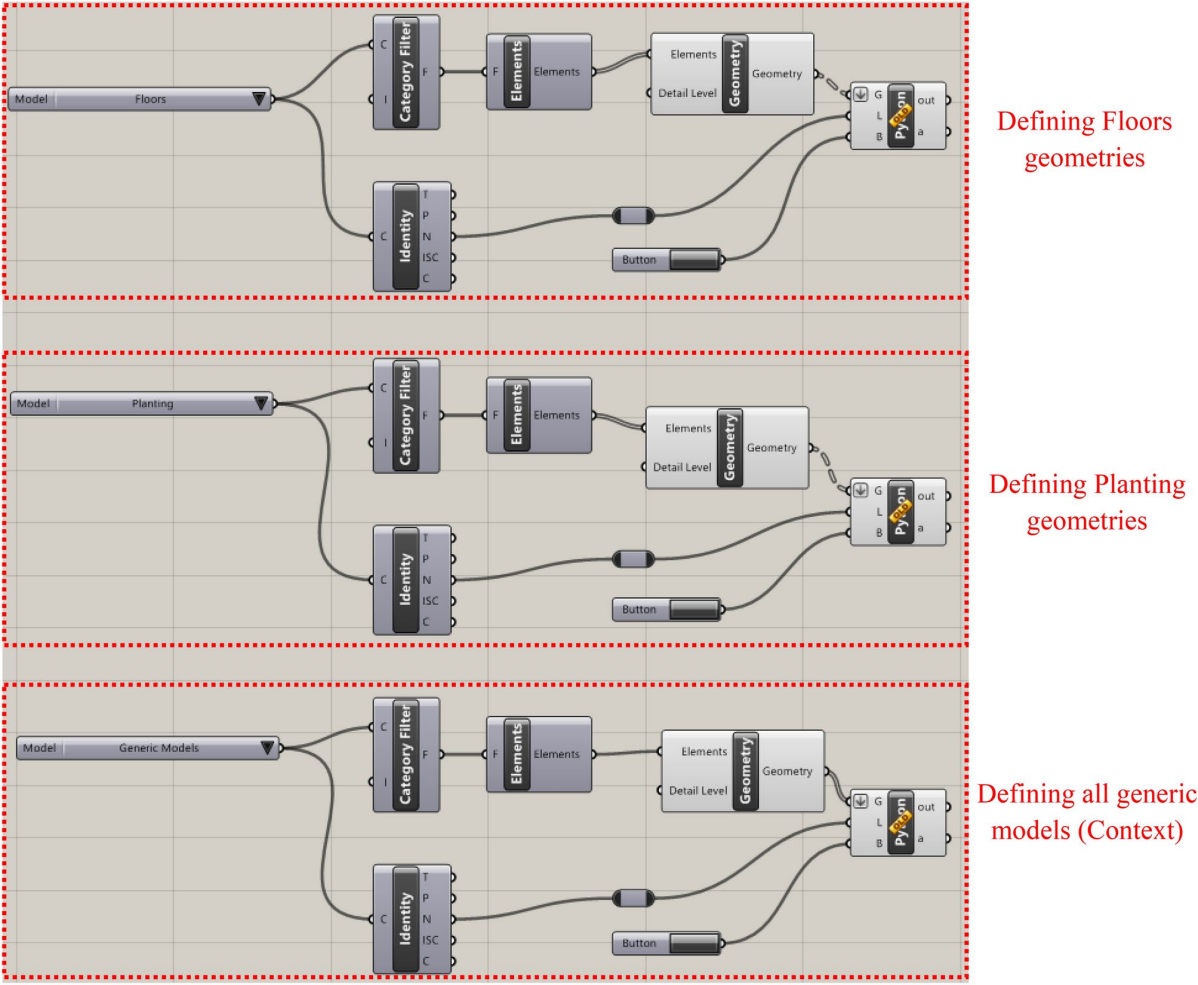
Revit to rhino visual script for identifying the model geometries by the Author.

Meanwhile, a visual script was created to identify each element inside the Revit models within its specific category. The three main elements to be synchronized are floors, planting, and generic models. (Fig. 39).

### UTCI analysis and calculations

**Fig. 40:**
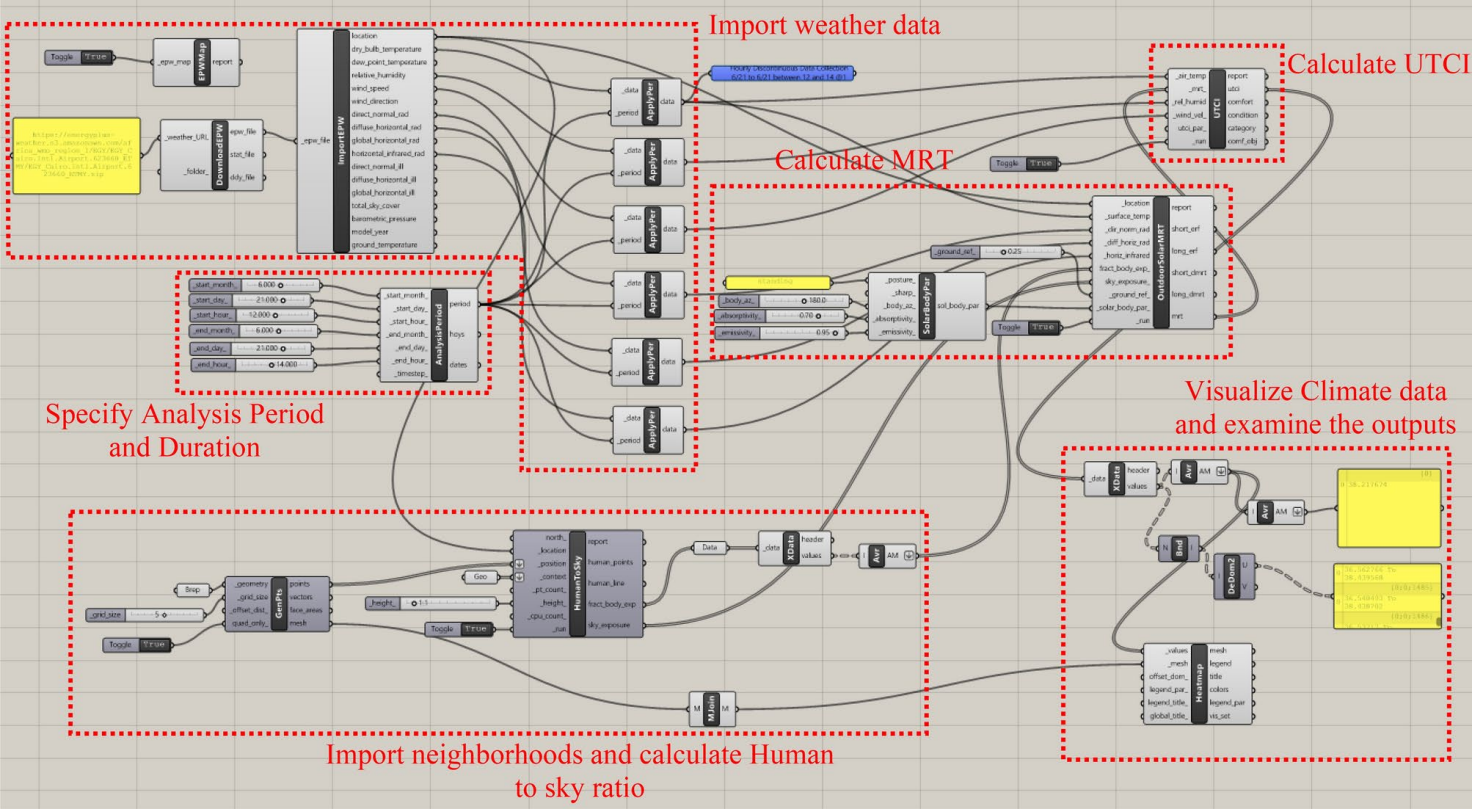
The full algorithmic script in the Grasshopper interface by the Author.

The Grasshopper interface was used to run the Ladybug tool. Over the past few years, multiple iterations of the Ladybug tool have been developed to improve the accuracy of measurements and simulated environments. Accordingly, the visual algorithmic script for the UTCI analysis was created using the latest version of the Ladybug tool, version 1.8.0, which was released on March 23, 2024^36^. The entire algorithmic script, including all the phases, is shown in (Fig. 40).

In the first Phase, the climate data for Cairo were imported using the EPW weather file^37^, and the required examination period was specified to match the simulation date and time of the previous simulations performed with Dynamo to ensure synchronization between all the results.

Second, the three neighborhoods with their six models (three base cases and three optimized cases) were imported, and their MRT and UTCI were calculated for a specific date and hour.

Finally, the difference between the base case scenarios and optimized scenarios was detected and recorded by calculating the arithmetic mean value of the heat stress for each case to assess the difference in outdoor thermal comfort and validate the generative design tool.

#### 1^st^ Phase, cairo climate data measurements

**Fig. 41:**
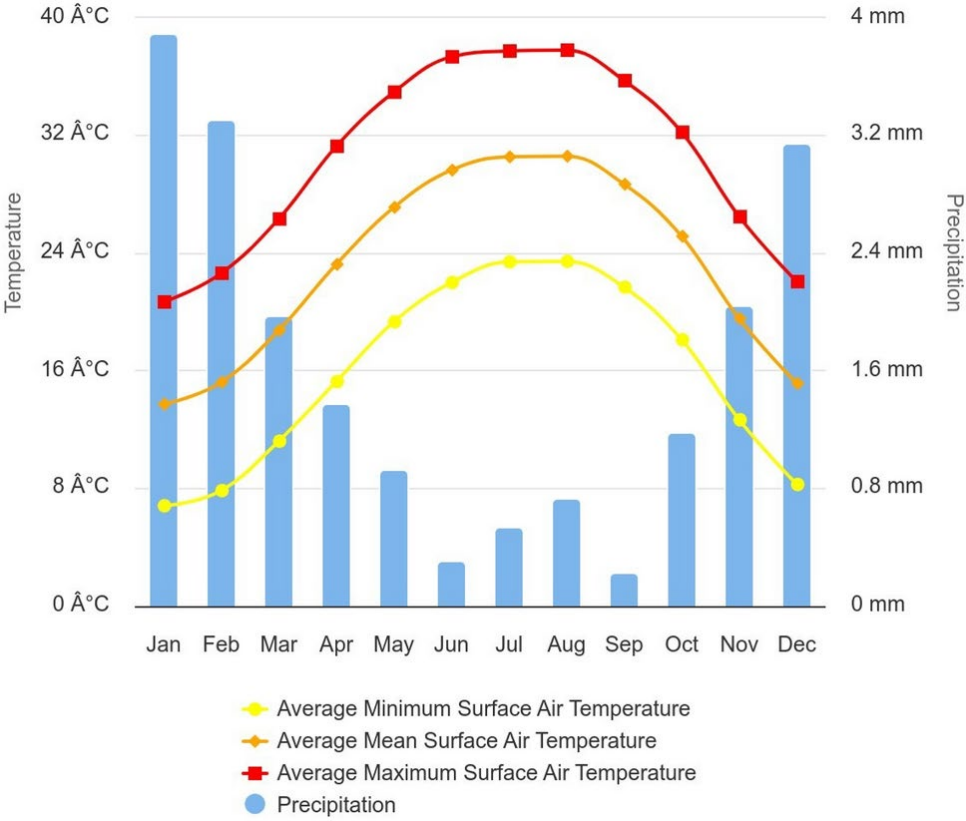
Monthly Climatology of Average Minimum Surface Air Temperature, Average Mean Surface Air Temperature, Average Maximum Surface Air Temperature & Precipitation 1991 - 2020; Arab Republic of Egypt by (World Bank Climate Change Knowledge Portal)^38^.

According to the 30-year metrological data obtained by the World Meteorological Data (WMO) station located at Cairo International Airport (30° 6’ 0’’ N, 31° 23’ 60’’ E), which is the nearest station to the selected neighborhoods June, July, and August were recorded as the hottest months across the entire year, and the maximum mean surface air temperature was recorded as shown in (Fig. 41)

- Import weather data and define the analysis period

The process starts by using the *"LB Download Weather"*component in order to convert the specified location’s URL into (.epw) and (.stat) weather files noting that all the meteorological data were recorded and obtained from Cairo International Airport station^39^. The (.epw) file was then directly plugged into the *“LB Import EPW”* component, which sequentially calculates the dry bulb temperature, wind speed, relative humidity, wind direction, diffuse horizontal radiation, direct normal radiation, and horizontal infrared radiation for the chosen location. These meteorological parameters are highly needed and prerequisites for calculating the MRT and UTCI. While, all of these components were connected to the analysis period node, which specified that the required duration was on the 21^st^ of June from 12:00 pm to 2:00 pm to match the optimized models and dynamo script.

b) Specify the analysis period and duration

Moreover, the *“LB Analysis Period”* component was used to specify the required period in terms of month, day, and hours for the three cases. Therefore, it will be connected directly with the weather data node to get the proper outputs for the required duration (Fig. 40).

#### 2^nd^ Phase, UTCI calculation

- Import the site context and the surface geometries

In this phase, the model of each neighborhood was loaded into the grasshopper to accurately analyze and calculate the fraction body exposure and sky exposure, as they are considered the main two required inputs for measuring the MRT and consequently UTCI. The fraction body exposure, which is a number between 0 and 1, represents the percentage of the body exposed to direct sunlight; it does not account for the body’s self-shading. While sky exposure is a value between 0 and 1, it indicates the percentage of the sky vault that can be seen by an individual^40^.

**Fig. 42:**
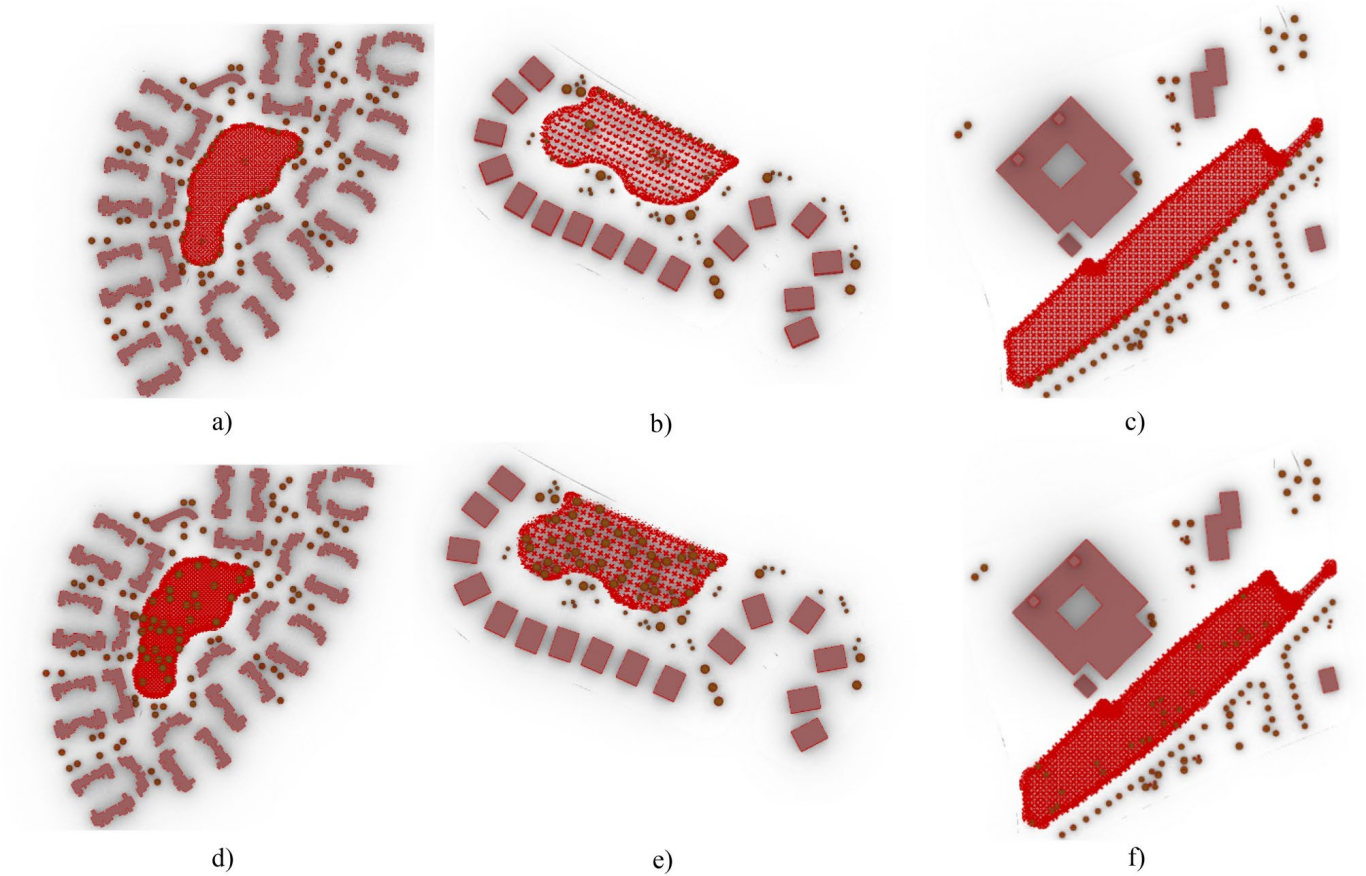
The generated grid from the “LB Human to Sky Relation” component for the 3 Base cases (Upside) and the 3 optimized cases (Downside), by the Author. **a** Clustered Neighborhood base case. **b** Semi-Clustered Neighborhood base case. **c** Open Neighborhood base case. **d** Clustered Neighborhood optimized case. **e** Semi-Clustered Neighborhood optimized case. **f** Open Neighborhood optimized case.

Accordingly, these two requirements can be estimated by using the *“LB Human to Sky Relation”* component by providing it with the location of each neighborhood, the position, and situation of residents in the outdoor environment, the surrounding context, the built environment that can block direct sunlight, and the height at which it required to be calculated. The location was obtained from the *“LB Import EPW”* component to determine the position and vertices of the residents, the *“LB Generate Point Grid”* component was used to generate the grid points on the required base by defining the surface boundary through using *“Brep”*and setting the grid size to five. While, for the surrounding built environment, buildings and trees were defined as context geometries that could prevent residents from being exposed to direct sunlight and impede their view of the sky vault. Lastly, the height value was set to 1.10 m (which is considered the height of the center of gravity of the human body) as per (Matzarakis, et al., 2014)^41^. Then by running the *“LB Human to Sky Relation”* component, a pointed grid was generated, with each point representing the location of the residents in the neighborhood setting. (Fig. 42)

b) Calculate mean radiant temperature (MRT)

The mean radiant temperature (MRT) is considered one of the main key factors in calculating thermophysiological comfort indexes (UTCI). It is the constant temperature of an imaginary environment setting where the radiant heat transfer from the human body is equivalent to that in the actual environment. It is a primary metric that monitors human energy balance, specifically on hot sunny days^42^.

The *“LB Outdoor Solar MRT”* component, which requires many inputs, was used to calculate the MRT. The first five inputs were from the *"LB Apply Analysis Period"* components, which combined the analysis period and climatic data. These inputs were (location, surface temperature, direct normal radiation, diffuse horizontal radiation, and horizontal infrared radiation). Then *“LB Human to Sky Relation”* component is subsequently used to provide the following two inputs (fraction body exposure and sky exposure).

**Fig. 43:**
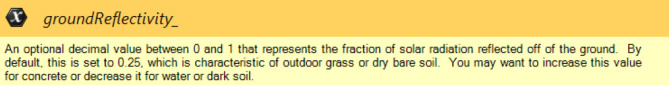
instruction of ground reflectivity in the grasshopper interface showing the albedo of outdoor grass 0.25.

The next needed input was ground reflectivity. The reflectance of a surface (albedo) is a value between 0 and 1. This value represents and outlines the percentage of solar energy reflected by a surface; 0 indicates the total absorption of solar energy, and 1 represents total reflectance. Surfaces with low albedo, such as asphalt and concrete, have a higher daytime temperature, whereas surfaces that have high albedo, such as grass, have lower temperature^43^. Moreover, the three selected neighborhoods have grassy open spaces. While grass has albedo values of 0.15 to 0.25^44^. However, the ground reflectivity in the Ladybug tool was set to a default value of 0.25 for outdoor grass or dry bare soil. (Fig. 43).

**Fig. 44:**
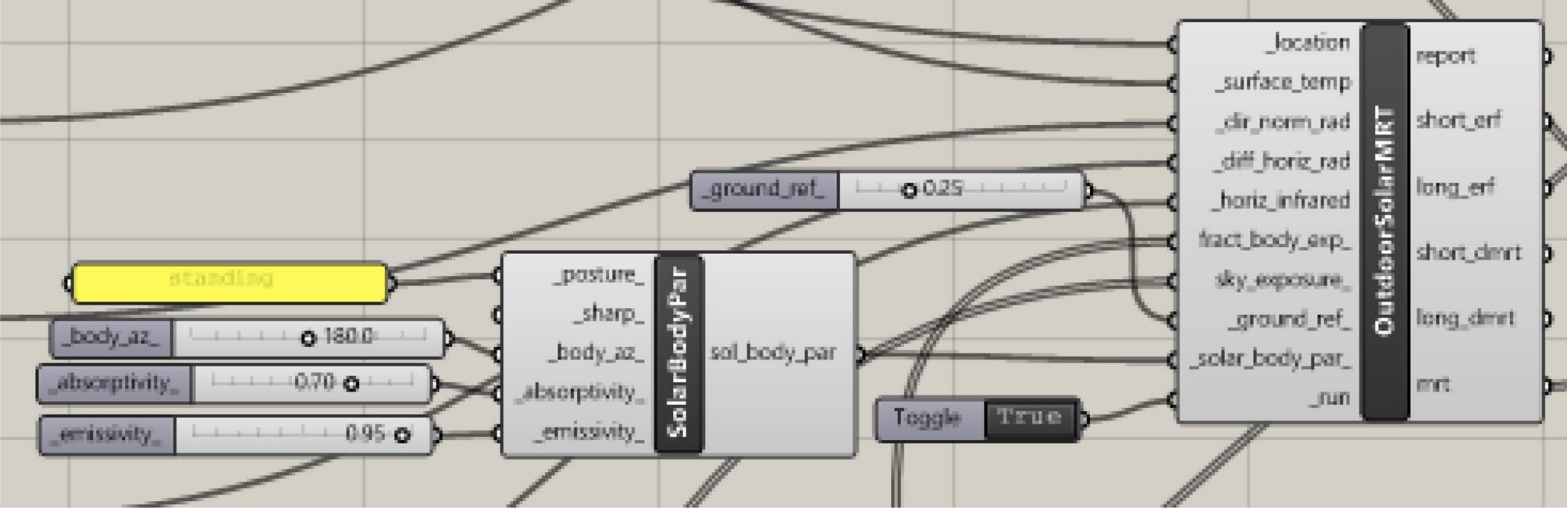
Solar Body Parameters plugged in MRT component.

The last input in the *“LB Outdoor Solar MRT”* component was solar body parameters, which are the assumptions of human geometry characteristics in the MRT computation. Accordingly, the *“LB Solar Body Parameters”* component was used to set these characteristics (Fig. 44); it requires four inputs:

1. The first input was“posture”, which was coded as standing; it can also be changed to seating or supine.
2. The second input was“Body Azimuth”, which is a value between 0° and 360° representing the orientation of the body in relation to the sun, in degrees, Accordingly, the azimuth angle was set assuming that the direction of the person is facing directly south which equals 180° and this is considered the extreme scenario.
3. The third was“absorptivity”, which is a value between 0 and 1, that represents the average shortwave absorptivity of the body, including clothing and skin color, whether white, brown, or black. The default variable was coded 0.7, which represents brown skin and medium clothing, therefore, the default variable was kept as it is.
4. The last input was“emissivity”, which is a value between 0 and 1, that represents the body’s average longwave emissivity. The default value is set to 0.95, which is nearly always the case except in some rare situations of wearing metallic or reflecting clothing.

Note that each of these inputs can be adjusted based on the site’s use and users. Finally, the output of these parameters was plugged into the *“LB Outdoor Solar MRT”* component as the last requirement then it was run to calculate the MRT.

c) Calculate and visualize the Universal Thermal Climate Index (UTCI).

**Fig. 45:**
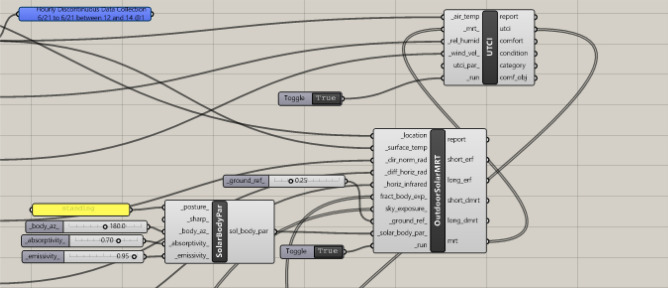
UTCI component.

UTCI was calculated by using the *“LB UTCI Comfort”* component, which requires four key parameters: air temperature, MRT, relative humidity, and wind velocity. The air temperature, relative humidity, and wind velocity data were obtained from the *“LB Apply Analysis Period”* component, which merged the climatic data with the chosen analysis duration in the first phase of the script. The MRT input was obtained from the *“LB Outdoor Solar MRT*” components. Finally, the *“LB UTCI Comfort”* component was used to calculate and run the UTCI. (Fig. 45).

Moreover, to visualize the UTCI results. Firstly, the *“LB Deconstruct Data”* component was utilized to filter out the values of the results, secondly, the average values “Athematic Mean” were extracted and plugged as input for the *“LB Spatial Heatmap”* component, which was used to visualize the calculated values that represent the average mean of UTCI values in the required duration in the three neighborhood scenarios (Existing scenario and optimized scenario).

## Results

The results show tangible positive outcomes in the three cases in terms of UTCI analysis, as detailed below in (Table 6):

**Table 6:** UTCI Evaluation for both cases (Base case and optimized case) in the three neighborhoods.

	**Vegetation counts in No.**	**UTCI arithmetic mean in celsius degree**	**UTCI maximum value in celsius degree**	**UTCI minimum value in celsius degree**
Clustered neighborhood (Base Case)	43	38.00 °C Very strong heat stress	39.49 °C Very strong heat stress	36.63 °C Strong heat stress
Clustered neighborhood (Optimized Case)	33	37.55 °C Strong heat stress	39.00 °C Very strong heat stress	35.60 °C Strong heat stress
Conclusion & differences	Optimized case is less by 10	The optimized case is lower by 0.45 °C.	The optimized case is lower by 0.49 °C.	The optimized case is lower by 1.03 °C.
Semi-clustered neighborhood (Base Case)	27	39.40 °C Very strong heat stress	40.09 °C Very strong heat stress	37.54 °C Strong heat stress
Semi-clustered neighborhood (Optimized Case)	45	38.01° C Very strong heat stress	39.63 °C Very strong heat stress	36.70 °C Strong heat stress
Conclusion & differences	Optimized Case is more by 18	The optimized case is lower by 1.39 °C.	The optimized case is lower by 0.46 °C.	The optimized case is lower by 0.84 °C.
Fully open neighborhood (Base Case)	31	39.60 °C Very strong heat stress	40.52 °C Very strong heat stress	39.23 °C Very strong heat stress
Fully open neighborhood (Optimized Case)	25	39.55 °C Very strong heat stress	40.60 °C Very strong heat stress	37.84 °C Strong heat stress
Conclusion & differences	Optimized Case is less by 6	The optimized case is lower by 0.05 °C.	The optimized case is higher by 0.08 °C.	The optimized case is lower by 1.39 °C.

**Fig. 46:**
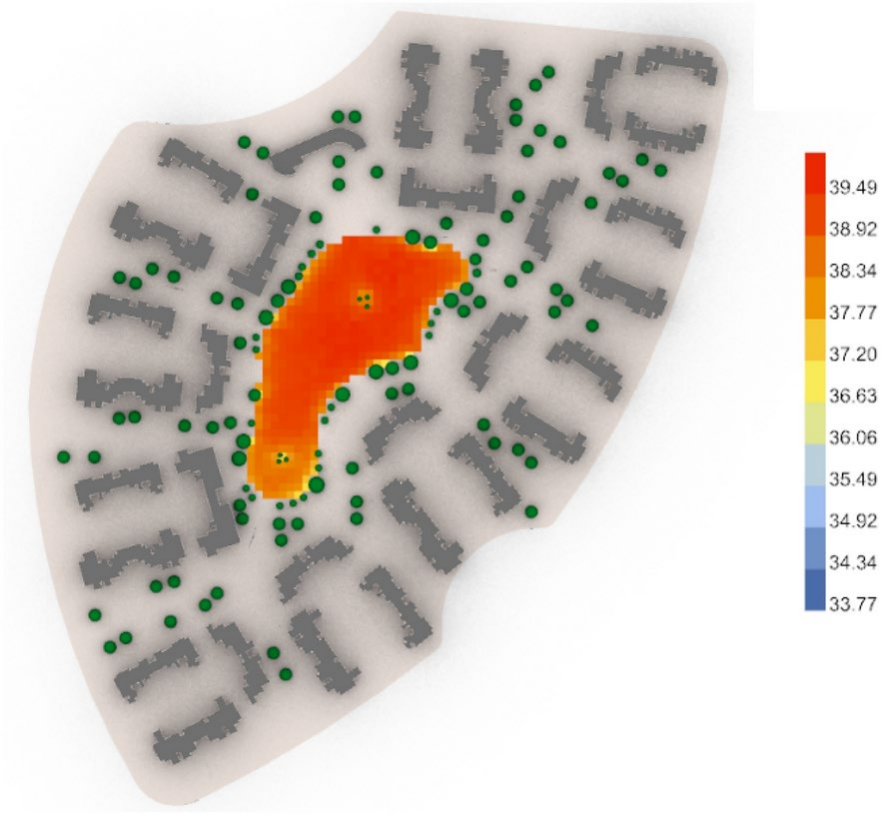
UTCI simulation for clustered neighborhood base case.

**Fig. 47:**
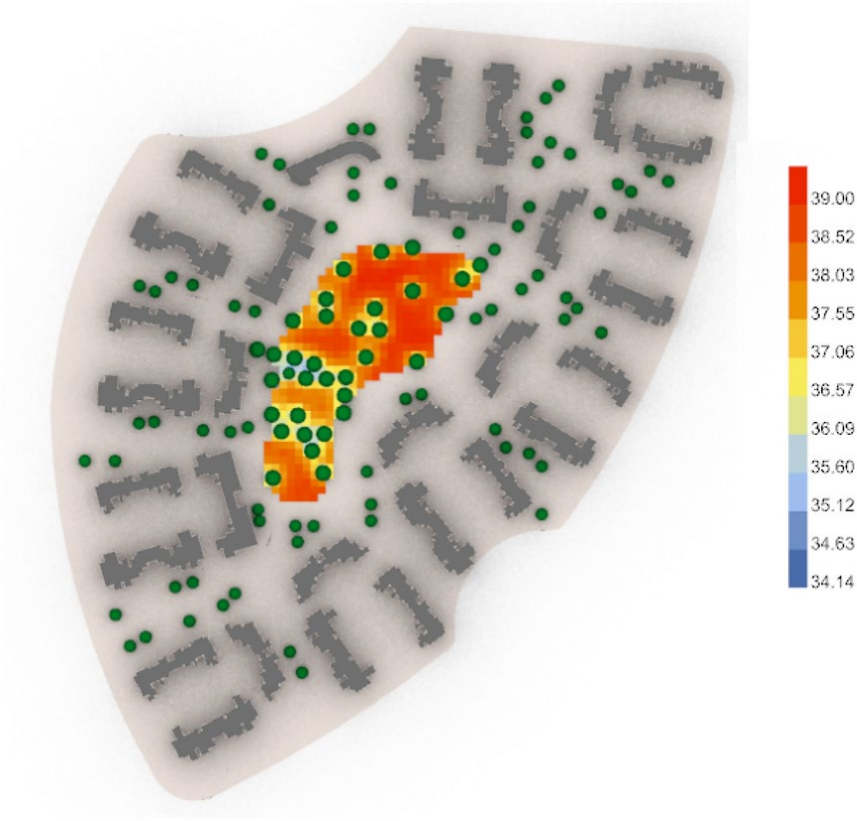
UTCI simulation for clustered neighborhood optimized case.
- For the second semi-clustered neighborhood, the base case (Fig. 48) gives an arithmetic mean value equal to 39.40 °C which is considered (Very strong heat stress) and gives 40.09 °C for maximum stress and 37.54 °C for the minimum stress with 27 vegetation counts, while the optimized case (Fig. 49) gives an arithmetic mean 38.01 °C which is considered still (Very strong heat stress). However, the global value of thermal comfort is decreased by almost 1.39 °C and the maximum stress is 39.63 °C, whereas the minimum stress is 36.70 °C with 45 vegetation counts.

- First the fully clustered neighborhood, the base case (Fig. 46) gives an arithmetic mean value of 38.00 °C, which is considered"Very Strong Heat Stress"according to the equivalent temperature scale. It shows a maximum stress of 39.49 °C and a minimum stress of 36.63 °C with 43 vegetation counts. While the optimized case (Fig. 47) gives an arithmetic mean value of 37.55 °C, which is considered as a"Strong Heat Stress,"with a difference of 0.45 °C from the base case. The optimized results show a maximum stress of 39.00 °C and a minimum stress of 35.60 °C with only 33 vegetation counts. This finding indicates that outdoor thermal comfort was slightly enhanced, despite the optimized case having 9 fewer trees than the base case.

**Fig. 48:**
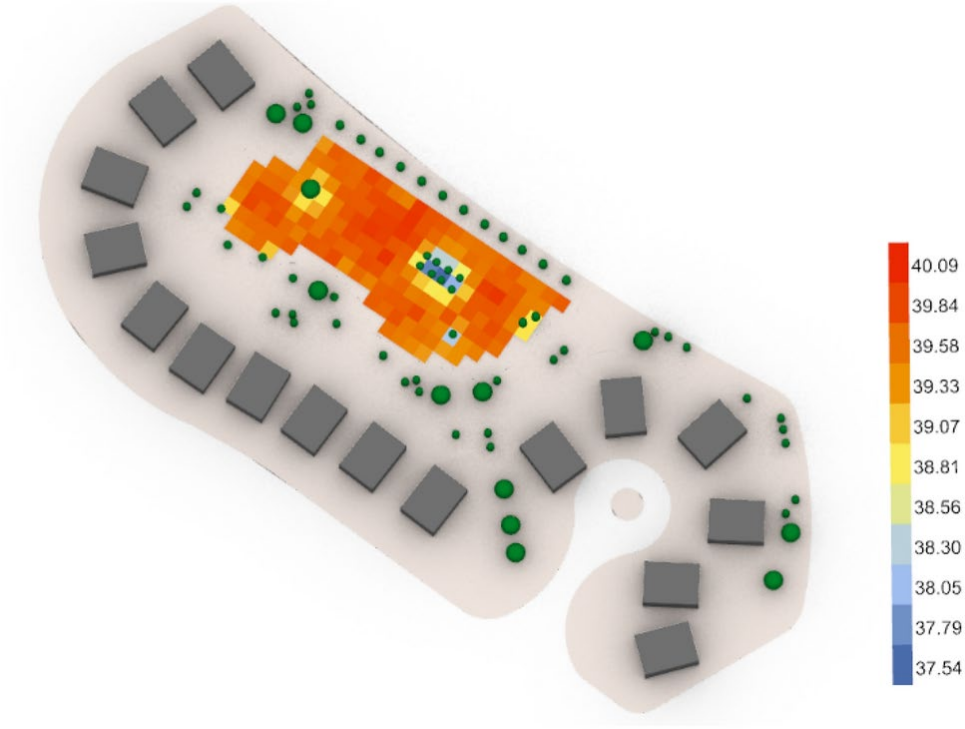
UTCI simulation for semi-clustered neighborhood base case.

**Fig. 49:**
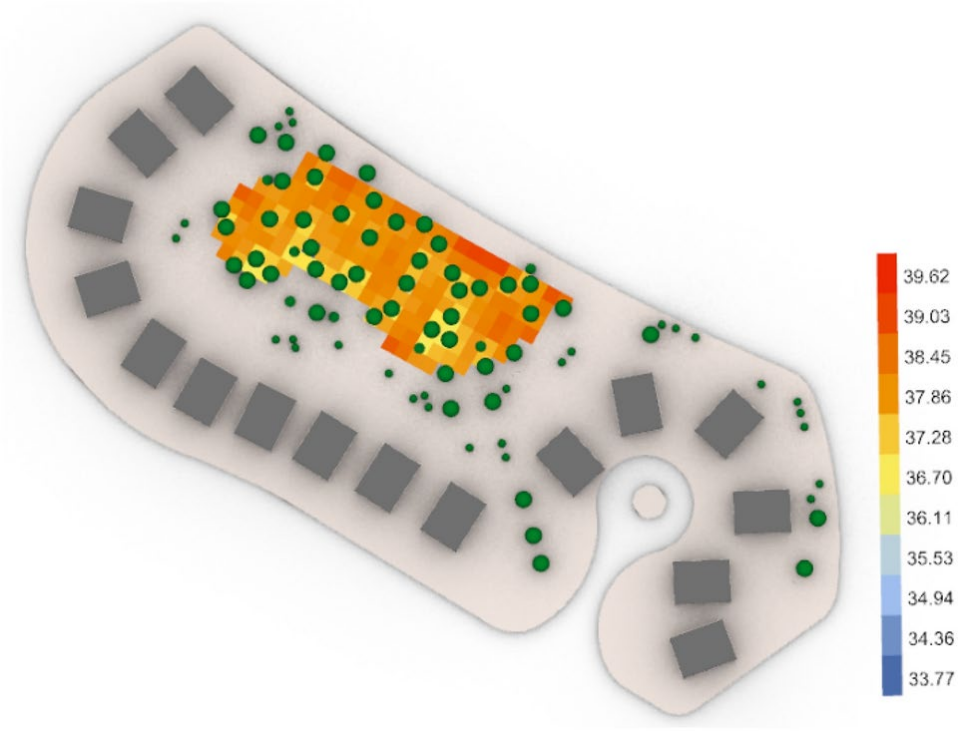
UTCI simulation for semi-clustered neighborhood optimized case.
- The third fully open neighborhood, the base case (Fig. 50) gives an arithmetic mean value 39.60 °C which is considered (Very strong heat stress) and gives 40.52 °C for the maximum stress and 39.23 °C for the minimal stress with 31 vegetation counts, while the optimized case (Fig. 51) gives an arithmetic mean value equal 39.55 °C which is considered (Very strong heat stress) with a difference from the base case almost 0.05 °C. Additionally, the results show a maximum stress of 40.60 °C and a minimum stress of 37.84 °C with only 25 vegetation counts, which means that the outdoor thermal comfort was enhanced very minorly, however, the palm count in the optimized case is less than that in the base case with 6 palms.

**Fig. 50:**
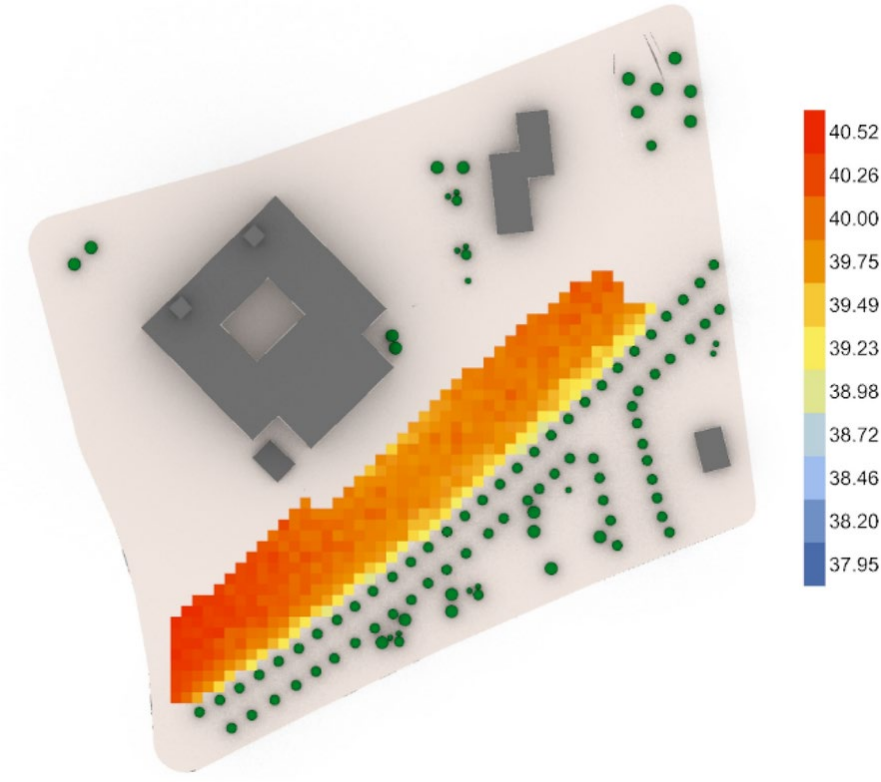
UTCI simulation for fully open neighborhood base case.

**Fig. 51:**
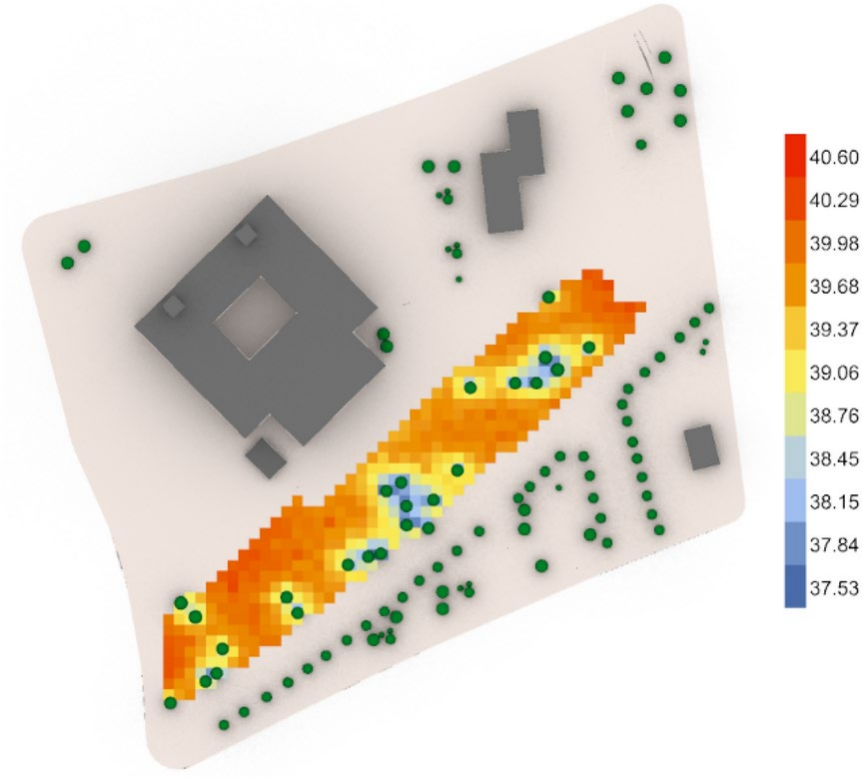
UTCI simulation for fully open neighborhood optimized case.

Nonetheless, these findings indicate a direct correlation between the distributions of trees/vegetation elements and outdoor thermal comfort. Accordingly, it is highly concluded that the generative design tool has the best performance in the clustered neighborhood while the least performance in the fully open neighborhood. This aligns with the findings of (Karimi et al., 2023)^28^, who demonstrated that vegetation-driven microclimatic interventions can effectively mitigate heat stress. Additionally, (Halder et al., 2022)^29^ emphasize that small-scale cooling measures contribute to lowering localized heat intensity, reinforcing the significance of the UTCI reductions observed in the study.

Despite this, the generative design tool can be considered a helpful tool in the pre-concept phases of landscape design, supporting landscape designers in obtaining a proper general overview of softscape design strategies in relation to outdoor thermal comfort. This computational method focuses on optimizing the vegetation counts while enhancing comfort, which in turn optimizes the project costs and water usage.

## Discussion

This framework model was created in accordance with a constraint-based design strategy and restraining parameters. Nonetheless, it was developed to examine the capabilities and potential of generative design tools for effectively optimizing tree distribution and softscape design based on environmental aspects through using Revit® and Dynamo as BIM tools, in addition to parametric design tools (Grasshopper®) with the aid of visual scripting language and environmental performance simulation plugins such as Ladybug©, which serve as assessment tool to evaluate the feasibility of using generative design tools during the pre-concept design stages.

In light of this, this research proposed a design solver strategy and framework, that shows a tangible improvements in the three selected urban neighborhoods in terms of MRT and UTCI analysis, while using fewer vegetation counts, as demonstrated by the study and interpreted in the results. Also, the environmental strategies developed in this study, though derived from the Madinaty context, are grounded in universal thermal comfort principles of hot arid regions. Consequently, the generative design framework presented here is expected to be adaptable to other similar urban settings.

Although UTCI reductions may appear minimal, research suggests that even small improvements in thermal conditions significantly enhance outdoor usability and user’s comfort. (Karimi et al., 2023)^28^demonstrated that optimized vegetation arrangements mitigate urban heat stress, leading to increased social interaction and prolonged outdoor activity. Similarly, (Halder et al., 2022)^29^highlighted that targeted green infrastructure strategies contribute to localized cooling effects, reducing the severity of the urban heat island (UHI) phenomenon. (Narimani et al., 2022)^45^ further emphasized that strategic tree placement alters microclimatic conditions by enhancing shading and evapotranspiration rates, thus improving thermal comfort.

Beyond thermal benefits, optimizing tree distribution plays a crucial role in reducing irrigation demands and maintenance costs while preserving cooling efficiency. Accordingly, these results can yield meaningful environmental, economic, and social benefits.

## Conclusion

This research developed a methodology that uses generative design tools with constraint-driven optimization logic to enhance OTC through strategic tree distribution in communal spaces. The findings demonstrate that optimizing tree placement can lower UTCI values and improve thermal conditions, even with reduced vegetation counts. By integrating AI and genetic algorithms, the proposed framework adapts to various urban morphologies, offering a dynamic, iterative design process that addresses immediate thermal comfort while supporting long-term sustainable urban development by reducing heat stress and promoting outdoor usability.

The generative design method can be considered an effective solver for complex design dilemmas, providing a range of design solutions that are difficult to explore through traditional approaches. This capability offers landscape designers valuable insights beyond human cognitive limits, enabling them to select the most suitable design option based on their criteria and environmental objectives. Despite the slight UTCI improvements, even minor reductions in thermal stress can significantly enhance outdoor livability, encouraging longer outdoor stays and fostering community interactions. The results validate the potential of generative design tools as powerful decision-support systems in the early design stages, guiding landscape architects toward more resilient and thermally comfortable urban spaces. Future research can build on this framework by incorporating more microclimatic parameters and validating results through longitudinal field studies, further refining the tool’s applicability across diverse urban contexts.

## Limitations

Weather meteorological data were obtained from EnergyPlus EPW weather file datasets, which provide high-level inputs. While, for greater accuracy, empirical validation through onsite measurements and model calibration should be recorded and used as microclimate data input and to be plugged into the ladybug script. As suggested by (Karimi et al., 2023)^28^, this approach would enhance the reliability of the model and improve the results in future studies.

This study positions the generative design tool as a decision-support framework in the pre-concept design stages, where early tree distribution strategies can be explored based on environmental performance metrics. While the tool provides optimized configurations through computational simulations, its applicability in real-world scenarios requires further empirical validation. Future research might explore additional validation methods, including PET and PMV simulations, post-implementation user surveys, and long-term microclimatic monitoring to refine the accuracy of the optimization process and assess its real-world effectiveness across diverse urban contexts.

Although this study examines only three neighborhood settings within Madinaty, Cairo, Egypt, the optimization framework is based on fundamental microclimatic principles applicable to all arid climates. While future research can apply this methodology to diverse urban contexts to further validate its generalizability.

Furthermore, while this study evaluates thermal comfort improvements through simulation indices such as UTCI and MRT, it does not account for long-term ecological impacts, maintenance requirements, or shifts in urban microclimates over time. In particular, factors such as species-specific performance data and selection, canopy growth rates, and irrigation demands require further assessment, as different species exhibit varying shading efficiencies and evapotranspiration rates that influence long-term cooling effectiveness. Future studies should incorporate lifecycle analysis and maintenance considerations to ensure that optimized tree distributions remain effective and sustainable over time. Additionally, built environment parameters, including surface reflectance index (SRI) and material thermal properties, can be considered to enhance the generative design approach for a more holistic optimization of thermal comfort.

Lastly, the interpreted and presented optimization framework, although aimed to reduce vegetation count and improve resource efficiency, while a detailed economic analysis of planting, maintenance, and associated benefits (e.g., property value and energy savings) was not performed. Future studies might incorporate a comprehensive cost-benefit evaluation to further validate the practical implementation of the proposed design strategy.

## Data Availability

The datasets used and/or analysed during the current study available from the corresponding author on reasonable request.
